# Transcriptome and Phytochemical Analyses Provide New Insights Into Long Non-Coding RNAs Modulating Characteristic Secondary Metabolites of Oolong Tea (*Camellia sinensis*) in Solar-Withering

**DOI:** 10.3389/fpls.2019.01638

**Published:** 2019-12-27

**Authors:** Chen Zhu, Shuting Zhang, Haifeng Fu, Chengzhe Zhou, Lan Chen, Xiaozhen Li, Yuling Lin, Zhongxiong Lai, Yuqiong Guo

**Affiliations:** ^1^ College of Horticulture, Fujian Agriculture and Forestry University, Fuzhou, China; ^2^ Institute of Horticultural Biotechnology, Fujian Agriculture and Forestry University, Fuzhou, China

**Keywords:** *Camellia sinensis*, withering, transcriptome, long non-coding RNAs, secondary metabolites

## Abstract

Oolong tea is a popular and semi-fermented beverage. During the processing of tea leaves, withering is the first indispensable process for improving flavor. However, the roles of long non-coding RNAs (lncRNAs) and the characteristic secondary metabolites during the withering of oolong tea leaves remain unknown. In this study, phytochemical analyses indicated that total polyphenols, flavonoids, catechins, epigallocatechin (EGC), catechin gallate (CG), gallocatechin gallate (GCG), epicatechin gallate (ECG), and epigallocatechin gallate (EGCG) were all less abundant in the solar-withered leaves (SW) than in the fresh leaves (FL) and indoor-withered leaves (IW). In contrast, terpenoid, jasmonic acid (JA), and methyl jasmonate (MeJA) contents were higher in the SW than in the FL and IW. By analyzing the transcriptome data, we detected 32,036 lncRNAs. On the basis of the Kyoto Encyclopedia of Genes and Genomes analysis, the flavonoid metabolic pathway, the terpenoid metabolic pathway, and the JA/MeJA biosynthesis and signal transduction pathway were enriched pathways. Additionally, 63 differentially expressed lncRNAs (DE-lncRNAs) and 23 target genes were identified related to the three pathways. A comparison of the expression profiles of the DE-lncRNAs and their target genes between the SW and IW revealed four up-regulated genes (*FLS*, *CCR*, *CAD*, and *HCT*), seven up-regulated lncRNAs, four down-regulated genes (*4CL*, *CHI*, *F3H*, and *F3’H*), and three down-regulated lncRNAs related to flavonoid metabolism; nine up-regulated genes (*DXS*, *CMK*, *HDS*, *HDR*, *AACT*, *MVK*, *PMK*, *GGPPS*, and *TPS*), three up-regulated lncRNAs, and six down-regulated lncRNAs related to terpenoid metabolism; as well as six up-regulated genes (*LOX*, *AOS*, *AOC*, *OPR*, *ACX*, and *MFP2*), four up-regulated lncRNAs, and three down-regulated lncRNAs related to JA/MeJA biosynthesis and signal transduction. These results suggested that the expression of DE-lncRNAs and their targets involved in the three pathways may be related to the low abundance of the total polyphenols, flavonoids, and catechins (EGC, CG, GCG, ECG, and EGCG) and the high abundance of terpenoids in the SW. Moreover, solar irradiation, high JA and MeJA contents, and the endogenous target mimic (eTM)-related regulatory mechanism in the SW were also crucial for increasing the terpenoid levels. These findings provide new insights into the greater contribution of solar-withering to the high-quality flavor of oolong tea compared with the effects of indoor-withering.

## Introduction

Long non-coding RNAs (lncRNAs) are a class of RNA transcripts that are longer than 200 nt and have no obvious open reading frame (Zhu and Wang, 2012; Ng et al., 2013). They usually have structural features that are similar to those of mRNA, such as 5′-cap structures and poly-A tails. Because of their low expression levels, lncRNAs were previously thought to be background “noise” produced by RNA polymerase II during transcription (i.e., no biological functions) (Li et al., 2014; Yan et al., 2019). Advances in lncRNA research revealed that most of the transcribed regions produce lncRNAs, which may actually have abundant functions (Kim and Sung, 2012; Bai et al., 2014). Specifically, lncRNAs have important roles at the transcriptional (Huarte et al., 2010), post-transcriptional (Carrieri et al., 2012), and epigenetic levels (Heo and Sung, 2011) *via* diverse regulatory mechanisms, including chromatin modification, transcriptional activation, transcriptional interference, and splicing regulation. In plants, lncRNAs regulate several metabolic activities, including sugar metabolism, organic acid metabolism, and amino acid metabolism. Moreover, some lncRNAs with microRNA (miRNA)-binding sites can serve as endogenous target mimics (eTMs) that bind to miRNAs to minimize the inhibition of target gene expression (Wu et al., 2013). In *Arabidopsis thaliana*, the lncRNA *IPS1*, which is an eTM of ath-miR399, can inhibit the cleavage of *PHO2* by ath-miR399 (Franco-Zorrilla et al., 2007). Similarly, several lncRNAs function by interacting with miRNAs in other plant species (Hao et al., 2015; Wang et al., 2015; Deng et al., 2018; Chen et al., 2018a). Furthermore, nta-eTMX27 in *Nicotiana tabacum* (Li et al., 2015), osa-eTM160 in *Oryza sativa* (Wang et al., 2017a), lncRNA23468 in *Solanum lycopersicum* (Jiang et al., 2019), and LTCONS-00042843 in *Dimocarpus longan* (Chen et al., 2018a) are eTMs that modulate target gene expression by functioning as decoys for relevant miRNAs. The development of high-throughput sequencing and bioinformatics tools enabled the identification of many lncRNAs in several plant species, including model plants like *A. thaliana* (Yuan et al., 2016), *O. sativa* (Zhang et al., 2014; Wang et al., 2018a), and *Brachypodium distachyon* (Quattro et al., 2017), and also in horticultural plants such as *S. lycopersicum* (Zhu et al., 2015), *Cucumis sativus* (Hao et al., 2015), *Musa itinerans* (Liu et al., 2018a), *D. longan* (Chen et al., 2018a), and *Citrus sinensis* (Ke et al., 2019). Moreover, lncRNAs in plants help regulate secondary metabolism and stress responses (Li et al., 2015; Zhang et al., 2018; Liu et al., 2018a; Wang et al., 2019a). However, there has been relatively little research regarding the genome-wide identification and analysis of lncRNAs in tea plants. Moreover, to the best of our knowledge, there is no published study on lncRNA functions associated with secondary metabolism during tea production.

Tea (*Camellia sinensis*) is an important economic crop that originated in China. Oolong tea, which is a semi-fermented type of tea, is produced mainly in Fujian, Guangdong, and Taiwan provinces in China. Additionally, it is very popular among consumers because of its unique floral and fruity aroma and mellow taste. Its postharvest processing involves a series of biochemical reactions that may regulate the accumulation of various secondary metabolites to enhance the flavor quality and economic value of oolong tea.

Tea flavor is greatly influenced by the manufacturing process (Chen et al., 2011a; Gui et al., 2015; Han et al., 2016a; Zhou et al., 2017). Withering is the first indispensable step for improving flavors during the postharvest processing of oolong tea, black tea, and white tea. Moreover, withering is closely related to the subsequent tea processing steps and is important for the development of the unique aroma and taste of teas. An important process in the traditional oolong tea production method involves a solar-withering treatment based on the tea leaf condition. Additionally, solar-withering positively regulates the production of floral and fruity aromas, thereby improving the aroma quality index of oolong tea (Kobayashi et al., 1985; Fu et al., 2015). During the solar-withering process, fresh tea leaves gradually shrink and soften because of the spontaneous and slow dehydration, and the tea flavor compounds undergo biochemical changes, which may contribute to the unique mellow and rich taste of oolong tea. Taste and aroma are the most important factors influencing the sensory quality of oolong tea. The characteristic compounds associated with tea quality include an extensive array of secondary metabolites, among which flavonoids (e.g., flavanols, phenolic acids, and anthocyanins) (Balentine et al., 1997) are the most important tea components responsible for the bitterness and astringency of teas. Volatile compounds are also important tea components for evaluating the flavor quality of oolong tea. On the basis of their metabolic pathways, the major volatile compounds in tea can be divided into the following four classes: fatty acid derivatives, terpenoids, phenylpropanoids/benzenoids, and carotenoid-derived compounds (Yang et al., 2013; Zheng et al., 2016). The volatile sesquiterpenoids and monoterpenoids, which are the main terpenoid metabolites, produce very sweet and floral aromas (Yang et al., 2013). Additionally, the transcription level of specific genes and the abundance of the encoded tea flavor compounds change considerably during the tea-withering process (Wang et al., 2019b). Furthermore, jasmonic acid (JA) and methyl jasmonate (MeJA), which function as elicitors, improve plant aromas by promoting the production of volatiles (Wasternack and Hause, 2013; Shi et al., 2015; Yu et al., 2017; Li et al., 2019). In the past few decades, several studies have attempted to characterize the mechanism underlying aroma formation during tea production based on analyses of transcription and metabolite contents (Moon et al., 1996; Lin et al., 2013; Zeng et al., 2016; Han et al., 2016a; Hu et al., 2018). Nevertheless, the regulatory effects of lncRNAs on the formation of tea flavor compounds during the withering of tea leaves remain unclear, especially the important lncRNA functions related to the improvement of oolong tea quality during solar-withering.

The release of the tea genome (Xia et al., 2017; Wei et al., 2018) has enabled the identification of lncRNAs in tea plants. To investigate the mechanism mediating the lncRNA effects on tea flavor-related metabolic activities during the withering of oolong tea leaves, we systematically identified lncRNAs in fresh leaves (FL), indoor-withered leaves (IW), and solar-withered leaves (SW) *via* transcriptome sequencing. We analyzed the target genes regulated by these lncRNAs and functionally characterized the lncRNAs involved in tea flavor-related metabolic activities. These lncRNAs, which may serve as eTMs to help regulate miRNAs and mRNAs, were also predicted and identified. Additionally, the contents of tea flavor-related metabolites, JA, and MeJA were analyzed. The role of JA/MeJA biosynthesis and signal transduction during the withering of oolong tea leaves was also explored. To the best of our knowledge, this is the first report describing the genome-wide identification and characterization of lncRNAs involved in tea production. These analyses provide important insights into the transcriptional changes and regulatory relationships of lncRNAs and their targets during the withering process of oolong tea production. Moreover, the importance of solar-withering on the flavor quality of oolong tea was revealed, as was the relationship between tea flavor metabolites and the expression patterns of lncRNAs and their target genes.

## Materials and Methods

### Plant Materials, Sample Preparation, and Sensory Analyses

Eight-year-old tea plants (*Camellia sinensis* cv. Tieguanyin) were cultivated at Fujian Agriculture and Forestry University, Fuzhou, Fujian province, China (E 119°14′, N 26°05′). Fresh shoots and the first three leaves were collected from each tea plant. The tea leaves were equally divided into three batches, each weighing 2 kg. The first batch was collected without any processing. The second batch was subjected to solar-withering under sunlight for 45 min (temperature: 25 ± 2°C; relative humidity: 60 ± 5%; illumination intensity: 40,000 ± 1,000 lx; and leaf layer thickness: 1 cm). The third batch of tea leaves was evenly layered and exposed to indoor light for 45 min (illumination intensity: 100 ± 5 lx; the other parameters of the indoor-withering process were the same as those used for the solar-withering process). The fresh leaves (FL), indoor-withered leaves (IW), and solar-withered leaves (SW) were collected and immediately frozen in liquid nitrogen and stored at −80°C for subsequent analyses. Experiments were completed with three independent biological replicates, each comprising material from more than 10 randomly selected tea plants.

For the initial analysis of the taste and aroma, the FL, IW, and SW samples were freeze-dried for 24 h before undergoing a sensory evaluation as previously described (Wang et al., 2019c).

### Determination of Leaf Water, Total Polyphenol, Total Flavonoid, Catechin, Lignin, Volatile Compound, Jasmonic Acid, and Methyl Jasmonate Contents

In order to determine the related metabolites, three independent biological replicates for the above-mentioned samples (FL, IW, and SW) were used for following studies. The water content of each tea leaf sample was determined as previously described (Wang et al., 2019b). The total polyphenol content was determined according to a Chinese national standard method (GB/T 8313-2018). Moreover, the total flavonoid contents of the three tea leaf samples were extracted and detected with the aluminum chloride colorimetric method (Do et al., 2014). Additionally, 0.1 g each tea leaf sample was diluted with methanol for a final concentration of 100 μg/ml. The 2.0 ml diluted sample was added to the extraction solution (0.1 ml of 10% aluminum chloride and 0.1 ml of 0.1 mM of potassium acetate solution) and incubated at room temperature for 30 min. The absorbance of the solution was measured at 415 nm with an ultraviolet (UV) light spectrophotometer. The total flavonoid content was quantified as previously described (Do et al., 2014). The catechins were extracted from the tea leaf samples and quantified according to a published method (Tai et al., 2015). Specifically, the catechins were analyzed with the Waters 2695 high-performance liquid chromatography (HPLC) system equipped with a 2489 UV/Visible detector. The detection wavelength was set to 278 nm, and the column temperature was maintained at 25°C. The following authentic standards were purchased from Solarbio (Beijing, China): catechin (C), gallocatechin (GC), epicatechin (EC), epigallocatechin (EGC), catechin gallate (CG), gallocatechin gallate (GCG), epicatechin gallate (ECG), and epigallocatechin gallate (EGCG). The lignin content was determined as previously described (Wang et al., 2012). Each sample was analyzed in triplicate.

Volatiles were extracted from the FL, IW, and SW and analyzed as previously described (Guo et al., 2019a). A Clarus SQ 8 gas chromatograph–mass spectrometer (PerkinElmer, New York, NY, USA) and a TurboMatrix Headspace System (PerkinElmer) were used to detect the volatile compounds. Each sample was analyzed in triplicate. The gas chromatography–mass spectrometry analysis was performed based on the TurboMass 6.1 software (PerkinElmer), and the separated compounds were identified according to their retention index and the National Institute of Standards and Technology Mass Spectral Library. Ethyl decanoate was applied as an internal standard. The volatile compound contents in the FL, IW, and SW were quantified according to an established procedure (Guo et al., 2019a).

JA was extracted from the three tea leaf samples and analyzed as previously described (Li et al., 2019). Each extracted sample (10 µl) was analyzed with the Waters 2695 HPLC system equipped with a 2489 UV/Visible detector, and the eluted JA was detected at 230 nm. To analyze the MeJA content, 0.3 g FL, IW, and SW tea samples were immersed in 80% ethyl alcohol for 24 h. After a centrifugation at 12,000 × g for 10 min, the supernatant was filtered through a 0.22-μm organic membrane. Each sample (10 µl) was then analyzed with the HPLC system equipped with the UV/Visible detector. The detection wavelength was set to 210 nm, and the column temperature was maintained at 30°C. Each sample was examined in triplicate. Authentic JA and MeJA standards were purchased from Solarbio (Beijing, China) and added to the extracts as internal standards. The JA and MeJA contents were quantified by calculating the area of each individual peak relative to the peak area of the authentic standards.

### Total RNA Extraction, Library Construction, and High-Throughput Sequencing

For high-throughput sequencing, total RNA was extracted from the FL, SW, and IW with the TransZol UP Reagent (TransGen Biotech, Beijing, China). The integrity of the purified RNA was assessed by gel electrophoresis and microvolume ultraviolet spectrophotometry (NanoDrop, Wilmington, DE, USA). Ribosomal RNA (rRNA) was eliminated from the purified RNA with the Ribo-Zero rRNA Removal Kit (Illumina, San Diego, CA, USA). Strand-specific cDNA was then synthesized to construct nine sequencing libraries (i.e., three biological replicates for the FL, IW, and SW tea samples) with the TruSeq Stranded Kit (Illumina), DNA polymerase I, and ribonuclease H. Each library was sequenced (paired-end reads) with the Illumina HiSeq X Ten platform (BGI, Shenzhen, China). All sequencing data were deposited in the National Center for Biotechnology Information (NCBI) Sequence Read Archive (accession number PRJNA562623).

### Transcriptome Assembly and Long Non-Coding RNA Identification

After removing the rRNA, low-quality reads, adapter sequences, and contaminating reads, the remaining clean reads were aligned to the tea reference genome (Xia et al., 2017) with the hierarchical indexing for spliced alignment of transcripts (HISAT) software. The transcriptome of each sample was independently assembled with the StringTie program (Martin and Wang, 2011; Pertea et al., 2015). Gene expression levels were calculated and normalized based on the fragments per kilobase per million mapped reads (FPKM) value (Li and Dewey, 2011).

To assess the quality of the transcriptome assembly, transcripts that overlapped with known genes in the tea reference genome were discarded, as were the short transcripts (≤200 bp) and the transcripts with a low FPKM value (< 0.5). The candidate lncRNAs selected for further investigation were those that satisfied the following criteria: coding potential calculator (score < 0) (Kong et al., 2007), txCdsPredict (score < 500) (Han et al., 2016b), coding-non-coding index (score < 0) (Sun et al., 2013a), and the transcript cannot be aligned to sequences in the Pfam database (El-Gebali et al., 2018). Moreover, the transcripts without a known genome annotation were reconstructed with the StringTie software. The reconstructed transcripts were then aligned to the reference annotation with the Cufflinks tool (Trapnell et al., 2012). The annotated transcripts were designated as MTCONS_ID.

### Analysis of Differentially Expressed Long Non-Coding RNAs and Their Targets

Differentially expressed lncRNAs (DE-lncRNAs) and differentially expressed genes (DEGs) were detected with the DEGseq software (Wang et al., 2009). The DE-lncRNAs and DEGs were identified based on the following criteria: |fold change| ≥ 2 and false discovery rate ≤ 0.001. Additionally, the Pearson correlation coefficient (PCC) and Spearman correlation coefficient (SCC) were used to determine the correlation between lncRNAs and their target genes (|PCC value| ≥ 0.6 and |SCC value| ≥ 0.6). A value greater than 0 indicated that the lncRNA was positively correlated with the target gene, whereas a value less than 0 indicated a negative correlation between the lncRNA and the target gene. Moreover, the regulatory effect of the lncRNAs on their target genes was designated as *cis*-acting or *trans*-acting based on the distance between the lncRNAs and their target genes (Knauss and Sun, 2013; Kornienko et al., 2013). Specifically, lncRNAs located 100 kb upstream or downstream of their target genes were defined as *cis*-acting, whereas lncRNAs located beyond this range were identified as *trans*-acting according to the binding energy (value ≤ 30) of the lncRNAs to the mRNAs. To clarify the potential functions of DE-lncRNAs and their target genes, the target genes were used as queries to screen the nonredundant protein (NR), Swiss-Prot, gene ontology (GO), and Kyoto Encyclopedia of Genes and Genomes (KEGG) databases with the BLAST algorithm (Altschul et al., 1990) (*E*-value < 1.0E^−5^). In the KEGG enrichment analysis, a false discovery rate ≤ 0.01 was applied as the criterion for identifying significantly enriched pathways. The log_2_-transformed (FPKM + 1) values were used to normalize the expression levels of the lncRNAs and their target genes. The expression levels of the DE-lncRNAs and DEGs were visualized with the TBtools software on the basis of the normalized FPKM values (Chen et al., 2018b).

### Prediction of the Endogenous Target Mimics and Targets of MicroRNAs

To explore the relationships between lncRNAs and miRNAs, the lncRNAs functioning as eTMs of miRNAs during the tea-withering process were identified with the TAPIR software according to previously described criteria (Bonnet et al., 2010). Furthermore, some lncRNAs and genes may serve as miRNA targets and are directly regulated by miRNAs. The lncRNAs and genes targeted by miRNAs were predicted with a plant small RNA target analysis server (psRNATarget). The lncRNAs and genes with an expected value greater than 5 were filtered, and the remaining lncRNAs and genes were identified as potential targets of miRNAs. The miRNA dataset for the FL, IW, and SW was obtained *via* small RNA sequencing.

### Relative Expression Analyses of the Selected Long Non-Coding RNAs, Messenger RNAs, and MicroRNAs

The total RNA of the FL, SW, and IW was reverse transcribed to first-strand cDNA with the TransScript First-Strand cDNA Synthesis SuperMix (TransGen Biotech, Beijing, China) for the quantitative real-time polymerase chain reaction (qRT-PCR) analysis of mRNA and lncRNA expression. Additionally, first-strand cDNA for the qRT-PCR analysis of miRNA expression was synthesized with the TransScript miRNA First-Strand cDNA Synthesis SuperMix (TransGen Biotech). The qRT-PCR assays of mRNAs, miRNAs, and lncRNAs were conducted on the LightCycler 480 platform (Roche Applied Sciences, Basel, Switzerland) using the TransStart Tip Green qPCR SuperMix (TransGen Biotech). The qRT-PCR procedure and program were completed as previously described (Zhou et al., 2019a). The *glyceraldehyde-3-phosphate dehydrogenase* (*GAPDH*) and *β-actin* were used as reference controls for normalizing the mRNA and lncRNA expression levels. Additionally, the *5.8S ribosomal RNA* (*5.8S rRNA*) and *U6 small nuclear RNA* (*U6 snRNA*) were used to normalize the miRNA expression level. The relative expression levels were calculated according to the 2^−ΔΔCt^ method, and all qRT-PCR primers were designed with the automated primer design tool of the Tea Plant Information Archive (**Supplementary Table S1**) (Xia et al., 2019). All qRT-PCR analyses were performed with three biological replicates. Moreover, the correlation between miRNA and target gene expression was determined based on the PCC and SCC.

### Mapping of Messenger RNA Cleavage Sites With the Modified 5′ RNA Ligase-Mediated Rapid Amplification of cDNA Ends

To verify the miRNA cleavage sites, a modified 5′ RNA ligase-mediated rapid amplification of cDNA ends (RACE) (5′ RLM-RACE) experiment was performed with the First-Choice RLM-RACE Kit (Thermo Fisher Scientific, Carlsbad, CA, USA) as previously described (Jeyaraj et al., 2019). The primers used to verify the cleavage sites are listed in **Supplementary Table S1**.

### Reverse Transcription–Polymerase Chain Reaction Analysis of MicroRNAs

The reverse transcription (RT)-PCR analysis of miRNAs was completed according to a slightly modified version of a published method (Fu et al., 2006). The PCR program was as follows: 94°C for 4 min; 40 cycles of 94°C for 30 s, 60°C for 30 s, and 72°C for 12 s; and 72°C for 10 min. The PCR products were detected by agarose gel electrophoresis. The RT-PCR primers are listed in **Supplementary Table S1**.

### Statistical Analyses

All data are herein expressed as the mean ± standard deviation of three independent biological replicates. Group differences were determined by a one-way analysis of variance followed by Tukey’s *post hoc* test. Significant differences among various groups are indicated with different letters. Specifically, a lowercase letter represents a significant difference (*p* < 0.05), whereas an uppercase letter represents an extremely significant difference (*p* < 0.01). All data were analyzed with the SPSS 25 software.

## Results

### Analysis of Water, Total Polyphenol, Total Flavonoid, Catechin, Lignin, Volatile Compound, Jasmonic Acid, and Methyl Jasmonate Contents of Fresh Leaves, Indoor-Withered Leaves, and Solar-Withered Leaves

**Figure 1:**
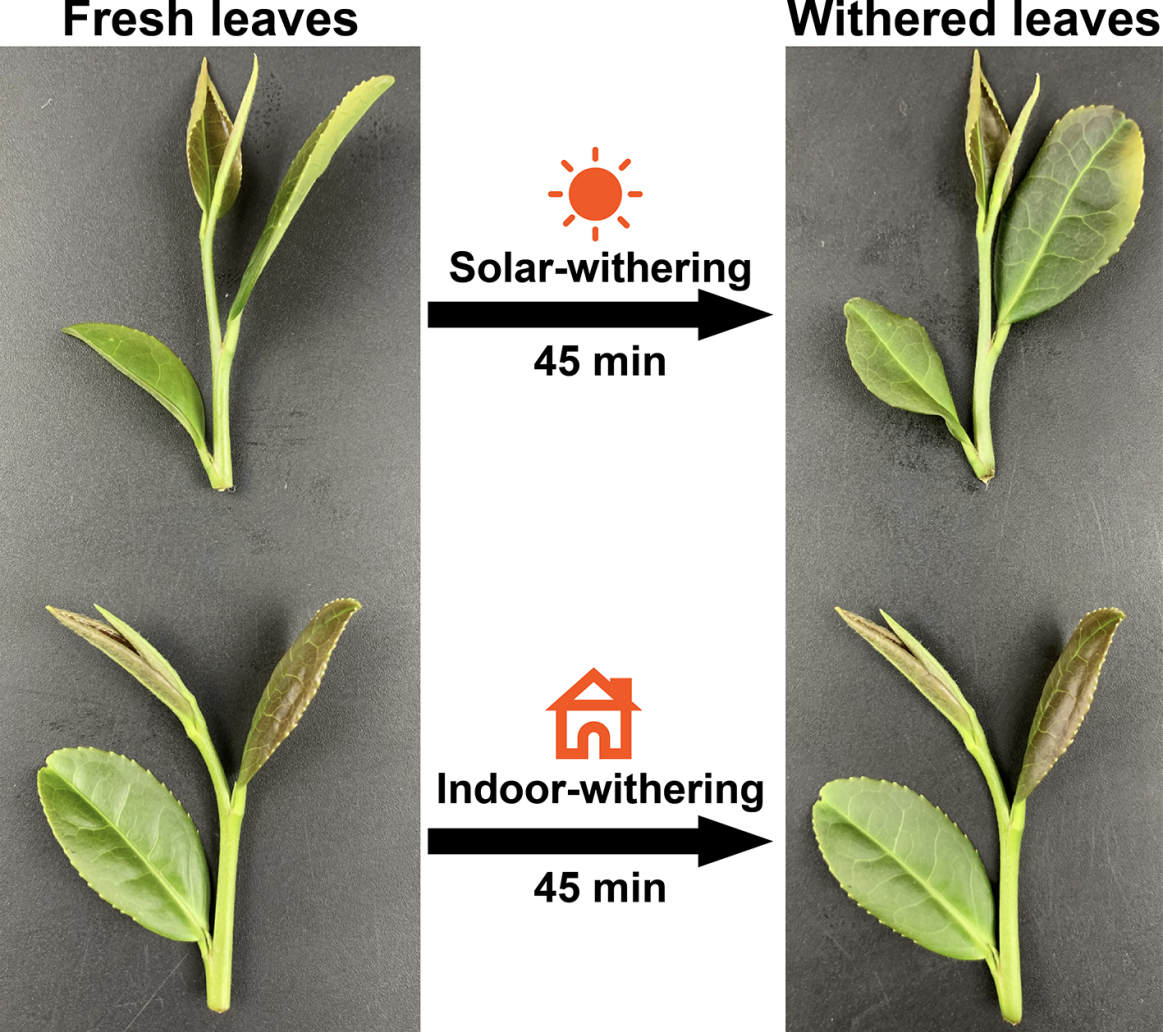
Leaf phenotype of fresh leaves, indoor-withered leaves, and solar-withered leaves.

The phenotypes of the FL, IW, and SW were recorded (**Figure 1**). The FL were glossy, straight, and green, with a water content of 75%. In contrast, the IW were slightly curled and deformed, with a water content of 68%. The SW were obviously curled and dull green. The appearance of the SW may have been due to their relatively low water content (65%). A sensory evaluation revealed that the three leaf samples differed regarding taste and aroma. Specifically, the FL and IW were bitter and astringent, with a strong grassy aroma, whereas the SW were weakly astringent, with a mellow taste, slightly grassy flavor, and faint floral and fruity aromas.

**Figure 2:**
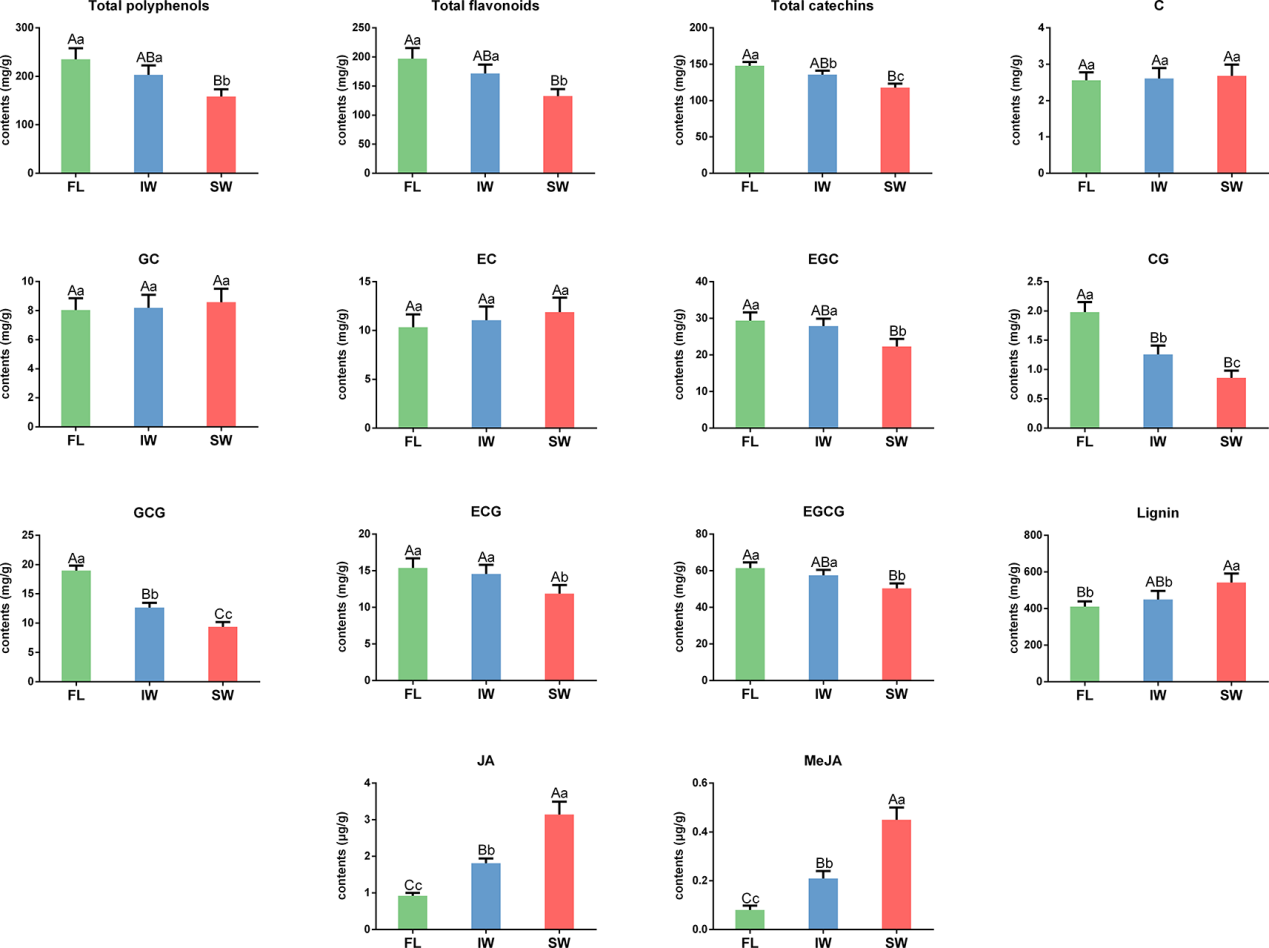
The contents of total polyphenols, total flavonoids, catechin, lignin, JA, and MeJA in fresh leaves (FL), indoor-withered leaves (IW), and solar-withered leaves (SW). C, catechin; GC, gallocatechin; EC, epicatechin; EGC, epigallocatechin; CG, catechin gallate; GCG, gallocatechin gallate; ECG, epicatechin gallate; EGCG, epigallocatechin gallate; JA, jasmonic acid; MeJA, methyl jasmonate. Error bars indicate standard deviation (SD) among three independent biological replicates. Data are presented as mean ± SD of three independent biological replicates. Lowercase letter indicates significant difference (*p* < 0.05); uppercase letter indicates highly significant difference (*p* < 0.01).

To explore the changes in the characteristic tea compounds in the FL, IW, and SW, we determined the contents of total polyphenols, total flavonoids, catechins, lignin, and volatiles in these samples. The total polyphenol contents were 235.47, 203.47, and 158.45 mg/g in the FL, IW, and SW, respectively (**Figure 2**). The total flavonoid content in the SW was significantly lower than that in the FL and IW. Similarly, the total flavonoid content was significantly lower in the SW (132.89 mg/g) than in the FL (197.20 mg/g) and IW (171.83 mg/g). Moreover, the abundance of total catechins was significantly lower in the SW than in the FL and IW. An analysis of individual catechins identified EGCG as the most abundant catechin in all three leaf samples. The EGCG content was significantly higher in the FL (61.37 mg/g) and IW (57.55 mg/g) than in the SW (50.35 mg/g). Similarly, EGC, CG, GCG, and ECG were significantly more abundant in the FL and IW than in the SW. However, there were no significant differences in the C, GC, and EC contents between the FL and SW, the FL and IW, and the IW and SW. Additionally, the lignin content was significantly higher in the SW (542.10 mg/g) than in the FL (410.18 mg/g) and IW (449.65 mg/g). Thus, the total flavonoid and total catechin contents as well as the abundance of five individual catechins, especially the galloylated catechins (CG, GCG, ECG, and EGCG), were significantly lower in the SW than in the FL and IW, whereas the opposite pattern was observed for the lignin content.

The contents of the top 20 volatiles in the FL, IW, and SW were detected by gas chromatography–mass spectrometry (**Table 1**). The fatty acid-derived volatile contents were significantly affected by withering. Analyses indicated that (*Z*)-3-hexenal and (*E*)-2-hexenal were the most abundant volatile components in the FL. After withering, the (*Z*)-3-hexenal and (*E*)-2-hexenal contents were significantly lower in the IW and SW than in the FL. A further analysis revealed that the (*Z*)-3-hexenal content of the SW (12.70%) was significantly lower than that of the IW (15.41%). Additionally, the (*E*)-2-hexenal content of the SW (12.20%) was significantly lower than that of the IW (16.92%). The abundance of terpenoid volatiles, including β-ocimene, limonene, γ-terpinene, α-farnesene, and β-myrcene, was significantly greater in the IW and SW than in the FL. A comparison of the terpenoid volatile contents in the IW and SW indicated that all five terpenoid volatiles were present at significantly higher levels in the SW than in the IW. Similarly, glycosidically bound volatiles (benzaldehyde, benzyl alcohol, phenethyl alcohol, and methyl salicylate) and a carotenoid-derived volatile (β-ionone) were more abundant in the IW and SW than in the FL. Therefore, the combined actions of various volatile compounds may be responsible for the formation of aromas during withering. Among these compounds, the (*Z*)-3-hexenal, (*E*)-2-hexenal, and terpenoid volatiles, which represent a large proportion of the volatiles in oolong tea, may be the most affected by the withering process, especially solar-withering, which can trigger a significant increase in the content of these terpenoid volatiles and a decrease in the abundance of hexenal volatiles.

**Table 1:** Relative contents of top 20 volatile compounds in FL, IW, and SW (%).

Volatile compounds	Retention time (min)	FL	IW	SW
**Fatty acid-derived volatile**				
(*Z*)-3-Hexenal	5.616	24.58 ± 2.12^Aa^	15.41 ± 1.58^Bb^	12.70 ± 1.22^Bc^
(*E*)-2-Hexenal	7.680	26.51 ± 2.39^Aa^	16.92 ± 1.66^Bb^	12.20 ± 1.21^Bc^
(*E*)-3-Hepten-1-ol	9.461	0.38 ± 0.03^Aa^	0.28 ± 0.02^Ab^	0.10 ± 0.01^Bc^
5-Methyl-2-hexene	10.274	2.27 ± 0.24^Aa^	2.10 ± 0.20^Aa^	1.34 ± 0.11^Bb^
(*E*)-3-Hexen-1-ol acetate	16.801	3.01 ± 0.33^Aa^	1.30 ± 0.13^Bb^	1.24 ± 0.12^Bb^
**Terpenoid volatile**				
β-Ocimene	21.058	0.17 ± 0.02^Cc^	0.46 ± 0.03^Bb^	0.88 ± 0.08^Aa^
Limonene	21.817	0.33 ± 0.04^Cc^	0.60 ± 0.07^Bb^	0.98 ± 0.09^Aa^
γ-Terpinene	22.413	7.65 ± 0.76^Bb^	8.53 ± 0.88^Bb^	10.56 ± 1.11^Aa^
α-Farnesene	22.988	0.49 ± 0.05^Bb^	0.62 ± 0.06^Bb^	1.18 ± 0.12^Aa^
β-Myrcene	23.454	2.01 ± 0.24^Bc^	2.61 ± 0.28^Bb^	3.79 ± 0.40^Aa^
**Glycosidically bound volatile**				
Benzaldehyde	10.773	0.36 ± 0.03^Bb^	0.47 ± 0.04^Bb^	0.99 ± 0.10^Aa^
Benzyl alcohol	14.570	0.06 ± 0.01^Bb^	0.09 ± 0.02^Bb^	0.79 ± 0.12^Aa^
Phenethyl alcohol	18.956	0.55 ± 0.05^Bc^	0.81 ± 0.08^Bb^	1.21 ± 0.13^Aa^
Methyl salicylate	25.040	0.44 ± 0.05^Bb^	0.77 ± 0.08^Aa^	0.88 ± 0.09^Aa^
**Carotenoid-derived volatile**				
β-Ionone	30.378	0.02 ± 0.01^Cc^	0.27 ± 0.02^Bb^	0.56 ± 0.03^Aa^
**Other volatile**				
Dimethyl sulfide	1.854	2.37 ± 0.21^Aa^	1.34 ± 0.13^Bb^	1.26 + 0.10^Bb^
1-Penten-3-one	3.001	3.64 ± 0.37^Aa^	2.10 ± 0.20^Bb^	2.35 ± 0.22^Bb^
2-Ethylfuran	3.176	4.84 ± 0.52^Bb^	4.56 ± 0.52^Bb^	6.95 ± 0.70^Aa^
2-Octen-1-ol	12.312	0.45 ± 0.06^Bb^	0.53 ± 0.06^Bb^	0.72 ± 0.10^Aa^
2-Methylnaphthalene	28.261	0.02 ± 0.01^Ab^	0.03 ± 0.01^Ab^	0.33 ± 0.03^Aa^

Data of volatile contents are presented as mean ± standard deviation (SD) of three independent biological replicates; lowercase letter indicates significant difference (p < 0.05); uppercase letter indicates highly significant difference (p < 0.01). FL, fresh leaves; IW, indoor-withered leaves; SW, solar-withered leaves.

Phytohormones, especially JA and MeJA, function as signaling molecules that promote the synthesis of related volatiles (Dudareva et al., 2013; Zeng et al., 2017). To clarify the accumulation of JA and MeJA during the withering process, we determined the JA and MeJA contents in fresh and withered leaves. The JA contents in the FL, IW, and SW were 0.92, 1.81, and 3.14 μg/g, respectively. The JA content was significantly higher in the SW (3.14 μg/g) than in the FL (0.92 μg/g) and IW (1.81 μg/g). Similarly, the MeJA content was also significantly higher in the SW (0.45 μg/g) than in the FL (0.08 μg/g) and IW (0.21 μg/g). The differences in the JA and MeJA contents among the three leaf samples may be related to the accumulation of volatiles.

### Sequencing and Assembly of Transcriptome Data

To investigate the regulatory mechanism of lncRNAs during the withering of oolong tea leaves, three biological replicates of the FL, IW, and SW underwent a transcriptome sequencing analysis with the Illumina HiSeq X Ten platform. After removing rRNA, low-quality reads, adapter sequences, and contaminating reads, an average of 12.66 Gb of clean data were obtained per sample (**Supplementary Table S2**). All clean reads were aligned to the tea reference genome. A total of 82,077 transcripts were detected, including 32,036 lncRNAs and 50,041 mRNAs. An analysis of the length distribution and exon number for the lncRNAs and mRNAs indicated that most of the lncRNAs (72.36%) were shorter than 1,000 bp and only 3.94% were longer than 3,000 bp. In contrast, 40.46% of the mRNAs were 0–1,000 bp and 10.07% of the mRNAs exceeded 3,000 bp. Moreover, 68.22% of the lncRNAs contained only one exon, whereas 85.98% of the mRNAs comprised more than two exons. These observations are consistent with the findings of studies on maize (Li et al., 2014), kiwifruit (Tang et al., 2016), and longan (Chen et al., 2018a), which confirmed that lncRNAs contain fewer exons than the annotated mRNAs and that most plant lncRNAs contain one exon.

The transcriptome dataset revealed that 28,677 lncRNAs were expressed. Additionally, 26,740 expressed lncRNAs were identified in the FL, which was more than the 26,508 expressed lncRNAs in the IW and the 26,479 expressed lncRNAs in the SW. Further analyses of lncRNA expression indicated that the expression levels of most of the lncRNAs in the FL, IW, and SW were low (FPKM ≤ 1), with no more than 5% of the total number of lncRNAs detected as highly expressed (FPKM ≥ 10) (**Supplementary Figure S1**).

### Identification of Differentially Expressed Long Non-Coding RNAs and Their Target Genes During the Tea-Withering Process

To identify the DE-lncRNAs during the tea-withering process, we analyzed the following comparisons: FL vs. IW, FL vs. SW, and IW vs. SW. The expression profiles based on the normalized FPKM values revealed 12,487 DE-lncRNAs in the FL, IW, and SW. Additionally 9,847 DE-lncRNAs were identified in the FL vs. IW comparison, of which 4,243 were up-regulated and 5,604 were down-regulated; 6,506 DE-lncRNAs were identified in the FL vs. SW comparison, of which 2,659 were up-regulated and 3,847 were down-regulated; and 4,964 DE-lncRNAs were identified in the IW vs. SW comparison, of which 2,443 were up-regulated and 2,521 were down-regulated.

The lncRNAs mediate the expression of some protein-coding genes through a *cis*- or *trans*-regulatory mechanism. Thus, the genes targeted by the DE-lncRNAs were predicted, which resulted in the identification of putative target genes for 2,892 DE-lncRNAs, including 2,180 *cis*-regulated and 1,096 *trans*-regulated target genes. Moreover, 1,694 DE-lncRNAs targeted only one protein-coding gene, whereas 14 DE-lncRNAs targeted more than six genes. A further analysis of the target genes revealed that 1,904 were regulated by only one DE-lncRNA, whereas 15 were regulated by more than six DE-lncRNAs.

### Functional Annotation of the Genes Targeted by Differentially Expressed Long Non-Coding RNAs Based on Gene Ontology and Kyoto Encyclopedia of Genes and Genomes Databases

The target genes were functionally characterized by aligning them to the sequences in the GO database with the BLAST algorithm. The 3,543 DE target genes in the FL vs. IW comparison were grouped into the three major GO categories (**Supplementary Figure S2**), namely, biological process, cellular component, and molecular function. The top three biological process subgroups were cellular process, metabolic process, and biological regulation. In the cellular component category, most of the DE target genes were annotated with the membrane, membrane part, and cell GO terms. Regarding the DE target genes in the molecular function category, most were classified into the following three subgroups: binding, catalytic activity, and transporter activity.

In the FL vs. SW comparison, 2,085 target genes were grouped into the three main GO categories (**Supplementary Figure S2**). The top three biological process subgroups (cellular process, metabolic process, and biological regulation) for the FL vs. SW comparison were consistent with the results for the FL vs. IW comparison.

Regarding the IW vs. SW comparison, only 890 target genes were annotated with the cellular process, metabolic process, and biological regulation GO terms (**Supplementary Figure S2**). In the biological process category, the three most enriched subgroups were cellular process, metabolic process, and response to stimulus. The top three enriched subgroups in the remaining two categories were consistent with the enrichment results for the FL vs. IW and FL vs. SW comparisons.

**Figure 3:**
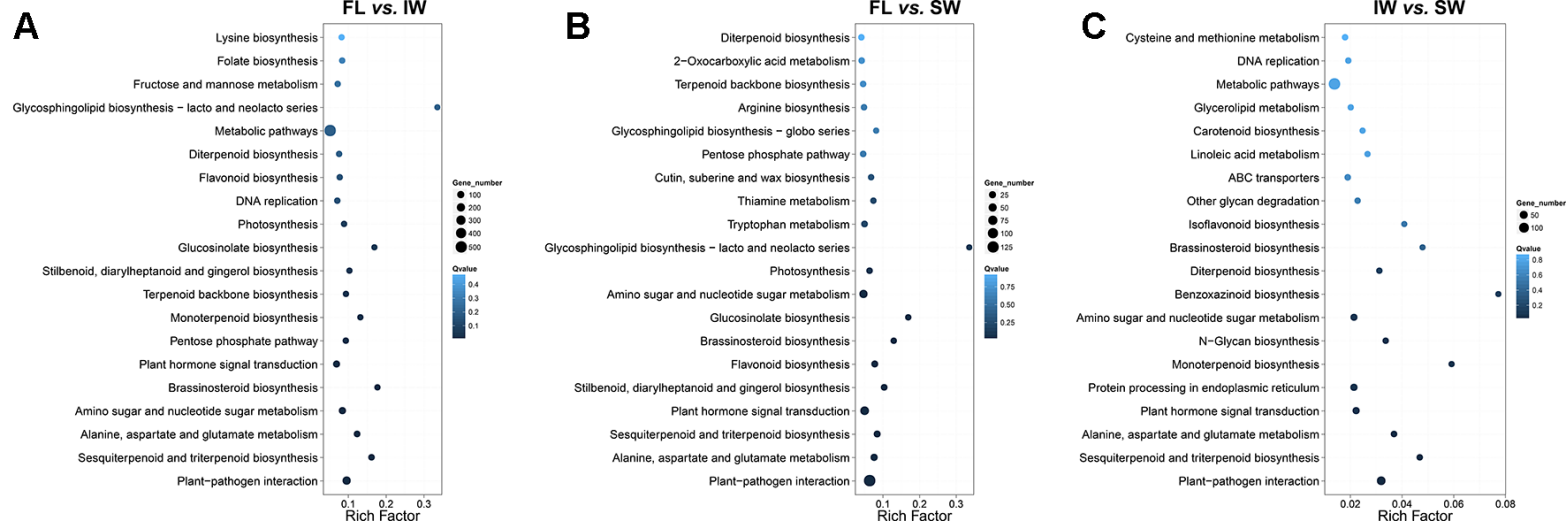
Kyoto Encyclopedia of Genes and Genomes (KEGG) enrichment analysis of the target genes of differentially expressed lncRNAs (DE-lncRNAs) identified in **(A)** FL vs. IW, **(B)** FL vs. SW, and **(C)** IW vs. SW. The higher rich factor represents more significant enrichment; the lower *q*-value represents more reliable enrichment.

We then identified the enriched KEGG pathways among the target genes of the DE-lncRNAs. In the FL vs. IW comparison, 3,543 DE target genes were enriched in 129 pathways (**Figure 3A**). The top 20 enriched pathways were as follows: plant–pathogen interaction; sesquiterpenoid and triterpenoid biosynthesis; alanine, aspartate, and glutamate metabolism; amino sugar and nucleotide sugar metabolism; brassinosteroid biosynthesis; plant hormone signal transduction; pentose phosphate pathway; monoterpenoid biosynthesis; terpenoid backbone biosynthesis; stilbenoid, diarylheptanoid, and gingerol biosynthesis; glucosinolate biosynthesis; photosynthesis; DNA replication; flavonoid biosynthesis; diterpenoid biosynthesis; metabolic pathways; glycosphingolipid biosynthesis; fructose and mannose metabolism; folate biosynthesis; and lysine biosynthesis.

In the FL *vs.* IW and FL *vs.* SW comparisons, seven of the top 20 enriched KEGG pathways were consistent (**Figure 3B**). These seven pathways were plant–pathogen interaction; plant hormone signal transduction; stilbenoid, diarylheptanoid, and gingerol biosynthesis; flavonoid biosynthesis; sesquiterpenoid and triterpenoid biosynthesis; glucosinolate biosynthesis; and monoterpenoid biosynthesis. Considering the effects of flavonoids and terpenoids on tea taste and aroma, respectively, we focused on the flavonoid and terpenoid metabolic pathways. The plant hormone signal transduction pathway is also involved, with linoleic acid metabolism related to the biosynthesis of JA and MeJA. Regarding plant hormone signal transduction, JA and MeJA induce the formation of terpenoid volatiles.

An analysis of the transcriptome differences between the IW and SW (**Figure 3C**) indicated that 13 of the top 20 enriched KEGG pathways were consistent between the FL vs. IW and FL vs. SW comparisons. The terpenoid metabolic pathways, including sesquiterpenoid and triterpenoid biosynthesis, monoterpenoid biosynthesis, and diterpenoid biosynthesis, were identified as crucial metabolic pathways. Additionally, the pathways related to flavonoid metabolism and plant hormone signal transduction also warrant further study.

### Analysis of the Differentially Expressed Genes and Differentially Expressed Long Non-Coding RNAs Involved in the Flavonoid Metabolic Pathway During Tea Withering

**Figure 4:**
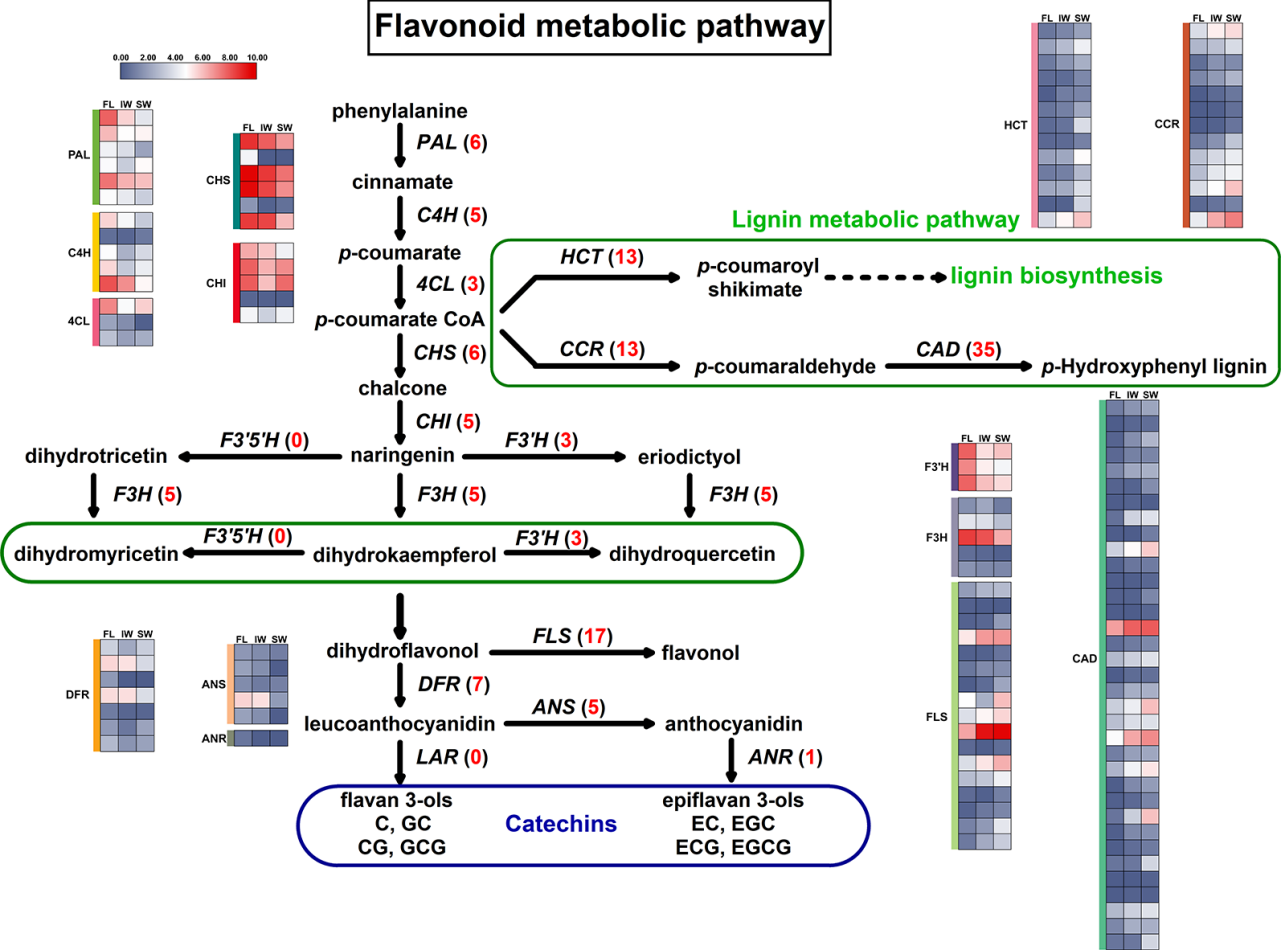
Differentially expressed genes (DEGs) involved in flavonoid metabolic pathway and lignin metabolic pathway. Numbers in parentheses following each gene name indicate the number of corresponding DEGs. The expression values of DEGs were normalized by log_2_-transformed (FPKM + 1).

The KEGG analysis identified the flavonoid metabolic pathway as one of the main enriched pathways. A total of 63 DEGs were assigned to flavonoid metabolic pathways. The following 11 gene families involved in flavonoid metabolic pathways were differentially expressed in the FL vs. IW, FL vs. SW, and IW vs. SW comparisons: *PAL* (*phenylalanine ammonia-lyase*), *C4H* (*cinnamate 4-hydroxylase*), *4CL* (*4-coumarate CoA ligase*), *CHS* (*chalcone synthase*), *CHI* (*chalcone isomerase*), *F3H* (*flavanone 3-hydroxylase*), *F3′H* (*flavonoid 3*′*-hydroxylase*), *FLS* (*flavonol synthase*), *DFR* (*dihydroflavonol 4-reductase*), *ANS* (*anthocyanidin synthase*), and *ANR* (*anthocyanidin reductase*) (**Supplementary Table S3**). Moreover, lignin and flavonoids are parallel secondary metabolites, and flavonoid biosynthesis and lignin biosynthesis may compete for the same substrates (Wang et al., 2017b). Thus, the following three lignin metabolism-related gene families were also identified: *CCR* (*cinnamoyl-CoA reductase*), *CAD* (*cinnamyl alcohol dehydrogenase*), and *HCT* (*hydroxycinnamoyltransferase*). A potential flavonoid metabolic pathway was constructed based on these identified DEGs (**Figure 4**).

The expression trends of 124 DEGs involved in the flavonoid and lignin metabolic pathways were compared based on the normalized FPKM values (**Figure 4**). All of the DEGs in six gene families (*C4H*, *4CL*, *F3H*, *F3′H*, *FLS*, and *HCT*) were more highly expressed in the FL and IW than in the SW. Additionally, most of the DEGs in six other gene families (*PAL*, *CHS*, *CHI*, *ANS*, *ANR*, and *DFR*) were also more highly expressed in the FL and IW than in the SW. Moreover, nine DEGs belonging to the *CCR* family were most highly expressed in the SW, as were more than half of the DEGs in the *CAD* family. Furthermore, 83 DEGs in the flavonoid metabolic pathway were more highly expressed in the IW than in the SW. These results implied that the flavonoid metabolism in the SW may have been in flux more weakly than that in the IW. Therefore, solar-withering may inhibit the accumulation of flavonoids in the withered leaves.

To decipher the regulatory mechanism of lncRNAs in flavonoid metabolism and lignin metabolism, the related DE-lncRNAs and their target genes were analyzed. A total of 31 DE-lncRNAs and eight DE target genes involved in the flavonoid and lignin metabolic pathways were identified in the FL, IW, and SW transcriptome dataset. The target genes included *4CL*, *CHI*, *F3H*, *F3’H*, *FLS*, *CCR*, *CAD*, and *HCT*. Additionally, the lncRNAs did not have a one-to-one regulatory relationship with the target genes. Specifically, 43 pairs of regulatory relationships (a lncRNA regulating an mRNA) were identified for these DE-lncRNAs and their target genes (**Supplementary Figure S3**). A correlation analysis indicated that 39 lncRNAs were positively correlated with target genes, whereas only four lncRNAs were negatively correlated with target genes (**Supplementary Table S4**). Moreover, the *HCT* and *CAD* genes were regulated by the most lncRNAs, with both gene families regulated by 10 lncRNAs. This was in contrast to *4CL*, *CHI*, *F3H*, and *F3’H*, which were regulated by only one lncRNA.

To further investigate the expression patterns of lncRNAs related to flavonoid and lignin metabolism, the expression patterns of 31 DE-lncRNAs were analyzed in the FL, IW, and SW based on the normalized FPKM values (**Supplementary Figure S4**). Similar lncRNA expression levels were detected for the FL and IW. Among the regulatory relationships between the lncRNAs and their target genes, 39 were positive regulatory relationships, whereas four were negative regulatory relationships. Moreover, we determined that some target genes were regulated by multiple lncRNAs and some lncRNAs simultaneously regulated multiple target genes. Thus, the lncRNAs may help regulate flavonoid contents by affecting the expression level of genes related to flavonoid metabolism *via* complex regulatory mechanisms.

**Figure 5:**
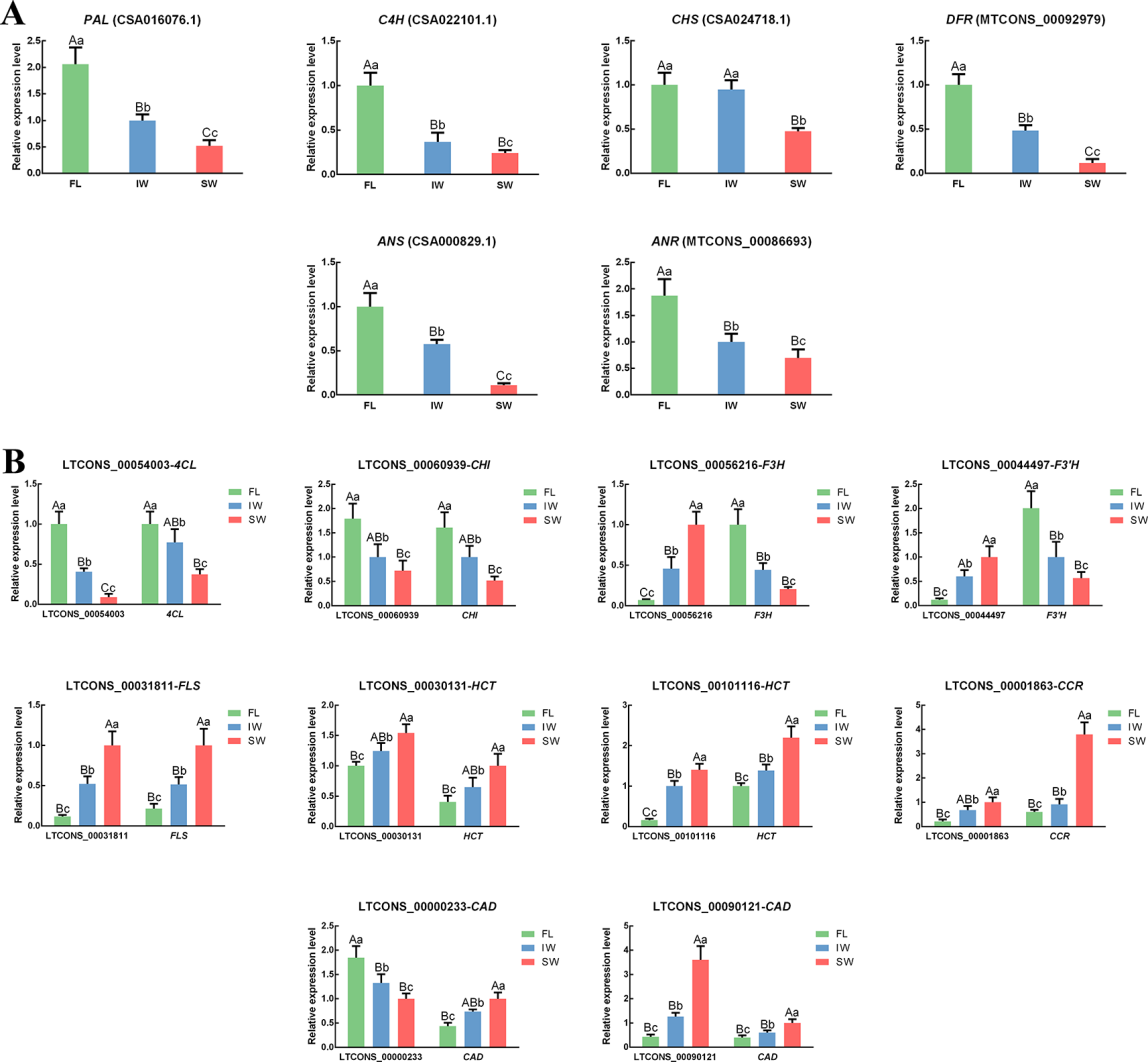
The expression patterns of **(A)** differentially expressed genes (DEGs), **(B)** differentially expressed lncRNAs (DE-lncRNAs) and their target genes in flavonoid metabolic pathway and lignin metabolic pathway determined by qRT-PCR. Data are presented as mean ± standard deviation (SD). Lowercase letter indicates significant difference (*p* < 0.05); uppercase letter indicates highly significant difference (*p* < 0.01).

To confirm the expression of the lncRNAs and their target genes in the transcriptome dataset, the expression patterns of 16 structural genes and 10 related lncRNAs involved in flavonoid metabolism in the FL, IW, and SW were analyzed by qRT-PCR (**Figure 5**). The correlation in the expression of the lncRNAs and their target genes was also analyzed (**Supplementary Table S5**). In the scaffolds, the structures of all important *cis*-regulated genes and their lncRNAs involved in flavonoid metabolism were plotted (**Supplementary Figure S5**). The following 10 lncRNAs were analyzed: LTCONS_00054003 (targeting *4CL*), LTCONS_00060939 (targeting *CHI*), LTCONS_00056216 (targeting *F3H*), LTCONS_00044497 (targeting *F3’H*), LTCONS_00031811 (targeting *FLS*), LTCONS_00001863 (targeting *CCR*), LTCONS_00000233 (targeting *CAD*), LTCONS_00090121 (targeting *CAD*), LTCONS_00030131 (targeting *HCT*), and LTCONS_00101116 (targeting *HCT*). The expression patterns of the detected lncRNAs and their target genes were consistent with the transcriptome dataset, implying that lncRNAs help regulate flavonoid metabolism by altering the expression levels of related genes. These expression-level changes may be an important factor mediating the obvious differences in the flavonoid metabolite contents between the IW and SW.

### Analysis of Differentially Expressed Genes and Differentially Expressed Long Non-Coding RNAs Involved in the Terpenoid Metabolic Pathway as Well as the Jasmonic Acid/Methyl Jasmonate Biosynthesis and Signal Transduction Pathway During Tea Withering

To characterize the expression of DEGs involved in the terpenoid metabolic pathway in the FL, IW, and SW, we examined the 59 DEGs associated with this pathway (**Supplementary Table S6**). Unlike the *DXR* (*1*-*deoxy*-*d*-*xylulose 5*-*phosphate reductoisomerase*), *MCT* (*2*-*C*-*methyl*-*d*-*erythritol 4*-*phosphate cytidylyltransferase*), *CMK* (*4*-*diphosphocytidyl*-*2*-*C*-*methyl*-*d*-*erythritol kinas*e), *GPPS* (*geranyl diphosphate synthase*), *PMK* (*phosphomevalonate kinase*), *MDC* (*mevalonate pyrophosphate decarboxylase*), and *FPS* (*farnesyl diphosphate synthase*) gene families, the following 11 gene families comprised more than two DEGs: *DXS* (*1*-*deoxy*-*d*-*xylulose*-*5*-*phosphate synthase*), *MDS* (*2*-*C*-*methyl*-*d*-*erythritol 2,4*-*cyclodiphosphate synthase*), *HDS* (*4*-*hydroxy*-*3*-*methylbut*-*2*-*enyl*-*diphosphate synthase*), *HDR* (*4*-*hydroxy*-*3*-*methylbut*-*2*-*enyl*-*diphosphate reductase*), *IDI* (*isopentenyl diphosphate isomerase*), *GGPPS* (*geranylgeranyl diphosphate synthase*), *AACT* (*acetyl*-*CoA C*-*acetyltransferase*), *HMGS* (*hydroxymethylglutaryl*-*CoA synthase*), *HMGR* (*hydroxymethylglutaryl*-*CoA reductas*e), *MVK* (*mevalonate kinase*), and *TPS* (*terpene synthase*).

**Figure 6:**
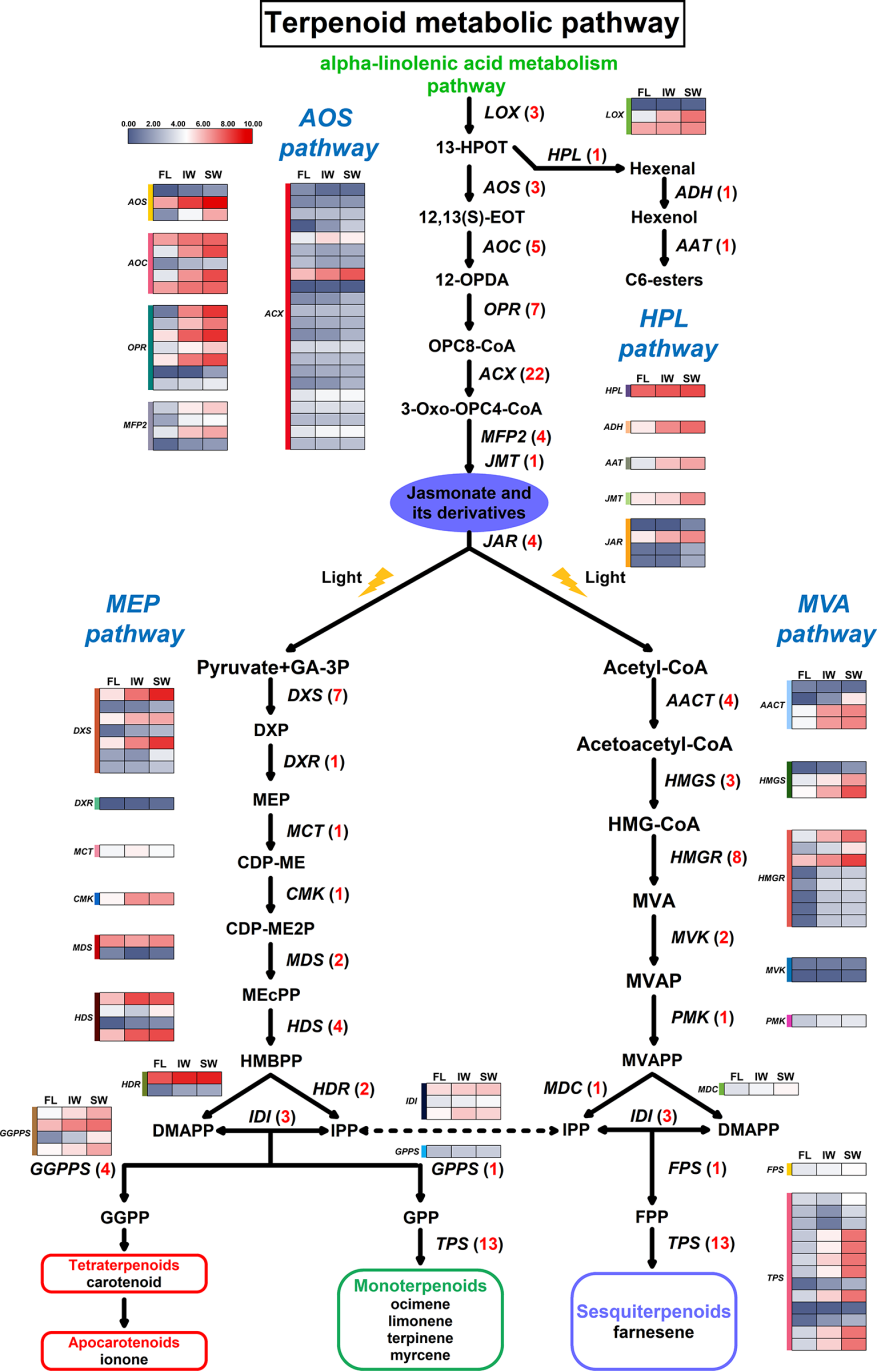
Differentially expressed genes (DEGs) involved in terpenoid metabolic pathway. Numbers in parentheses following each gene name indicate the number of corresponding DEGs. The expression values of DEGs were normalized by log_2_-transformed (FPKM+1).

An analysis of the JA/MeJA biosynthesis and signal transduction pathway uncovered 49 DEGs that were assigned to the following eight gene families: *LOX* (*lipoxygenase*), *AOS* (*allene oxide synthase*), *AOC* (*allene oxide cyclase*), *OPR* (*oxophytodienoic acid reductase*), *ACX* (*acyl*-*CoA oxidase*), *MFP2* (*enoyl*-*CoA hydratase*/*3*-*hydroxyacyl*-*CoA dehydrogenase*), *JMT* (*jasmonate O-methyltransferase*), and *JAR* (*jasmonic acid*-*amino synthetase*) (**Supplementary Table S7**). With the exception of the *JMT* family, these gene families included more than two DEGs, with the *ACX* family having the most DEGs. Moreover, changes in the hexenal content are related to the hydroperoxide lyase (HPL) pathway. This pathway along with the AOS pathway function downstream of the LOX pathway. Thus, we also analyzed three genes in the HPL pathway, namely, *HPL* (*fatty acid hydroperoxide lyase*), *ADH (alcohol dehydrogenase*), and *AAT (alcohol acetyltransferase*). A possible terpenoid metabolic pathway was constructed based on these identified DEGs (**Figure 6**).

Comparisons of the transcript levels (FPKM values) of these DEGs revealed that 17 DEGs were more highly expressed in the IW and SW than in the FL. Moreover, the *MDS* genes exhibited complex expression trends. On the basis of the normalized FPKM values, we also compared the expression trends of the 49 DEGs involved in the JA/MeJA biosynthesis and signal transduction pathway between the fresh and withered leaves (**Figure 6**). Most of the DEGs in these gene families were more highly expressed in the SW than in the IW. Specifically, all of the DEGs corresponding to *LOX*, *AOS*, *AOC*, *OPR*, *MFP2*, *JMT*, and *JAR* were most highly expressed in the SW. Additionally, the expression levels of 17 DEGs corresponding to *ACX* were also higher in the SW than in the IW. These results suggested that the high transcription levels for these six JA/MeJA biosynthesis-related genes in the SW may influence JA and MeJA accumulation. Moreover, *HPL*, *ADH*, and *AAT* were also most highly expressed in the SW, which may be related to hexenal content changes.

The high expression levels of most of the DEGs corresponding to the 17 gene families involved in the terpenoid metabolic pathway may be positively correlated with the terpenoid contents. Moreover, the high expression levels of eight gene families influencing the JA/MeJA biosynthesis and signal transduction pathway may promote the accumulation of JA and MeJA, which may induce the synthesis of terpenoid volatiles (Dudareva et al., 2013). Thus, these results suggested that the transcript levels of these DEGs may be related to the terpenoid contents in the FL, IW, and SW.

To further elucidate the regulatory effects of lncRNAs on the terpenoid metabolic pathway and the JA/MeJA biosynthesis and signal transduction pathway, we also predicted the DE-lncRNAs and their target genes related to these two pathways. The correlation analysis indicated that eight lncRNAs were positively correlated with the target genes, whereas 11 lncRNAs were negatively correlated with the target genes (**Supplementary Table S8**). Moreover, we identified 10 DE-lncRNAs affecting 14 *cis*-target genes and four DE-lncRNAs affecting five *trans*-target genes in the terpenoid metabolic pathway. A subsequent analysis revealed that *GGPPS* and *TPS* are regulated by three lncRNAs, whereas four genes (*DXS*, *CMK*, *AACT*, and *PMK*) are targeted by only one lncRNA (**Supplementary Figure S6**).

In the JA/MeJA biosynthesis and signal transduction pathway, 18 DE-lncRNAs and 18 DE target genes were identified in the FL, IW, and SW. Of the target genes, six associated with the JA/MeJA biosynthesis and signal transduction pathway (*LOX*, *AOS*, *AOC*, *OPR*, *ACX*, and *MFP2*) were regulated by lncRNAs. The correlation analysis demonstrated that 17 lncRNAs were positively correlated with the target genes, whereas five lncRNAs were negatively correlated with the target genes (**Supplementary Table S9**). We also clarified the specific lncRNA–mRNA regulatory relationships. A total of 22 pairs of lncRNA–mRNA regulatory relationships were detected among these DE-lncRNAs and target genes involved in the JA/MeJA biosynthesis and signal transduction pathway.

To analyze the relationship between the expression patterns of DE-lncRNAs and their target genes, we compared their expression levels in the FL, IW, and SW. The expression patterns of 32 DE-lncRNAs were analyzed in the FL, IW, and SW based on the normalized FPKM values (**Supplementary Figure S7**). The overall expression levels of the DE-lncRNAs were similar in the IW and SW but varied in the FL. Among the 41 lncRNA–mRNA regulatory pairs, 25 pairs comprised a lncRNA and potential target gene that exhibited the same expression trend, whereas 16 pairs consisted of a lncRNA and target gene that exhibited the opposite expression trend. An examination of the regulatory relationships between the lncRNAs and mRNAs detected eight lncRNAs that targeted more than one gene and 24 lncRNAs with only one target gene. These findings implied that most of the lncRNAs associated with these two pathways have a single regulatory function regarding the expression of target genes. However, the few lncRNAs that target several genes may have multiple functions.

**Figure 7:**
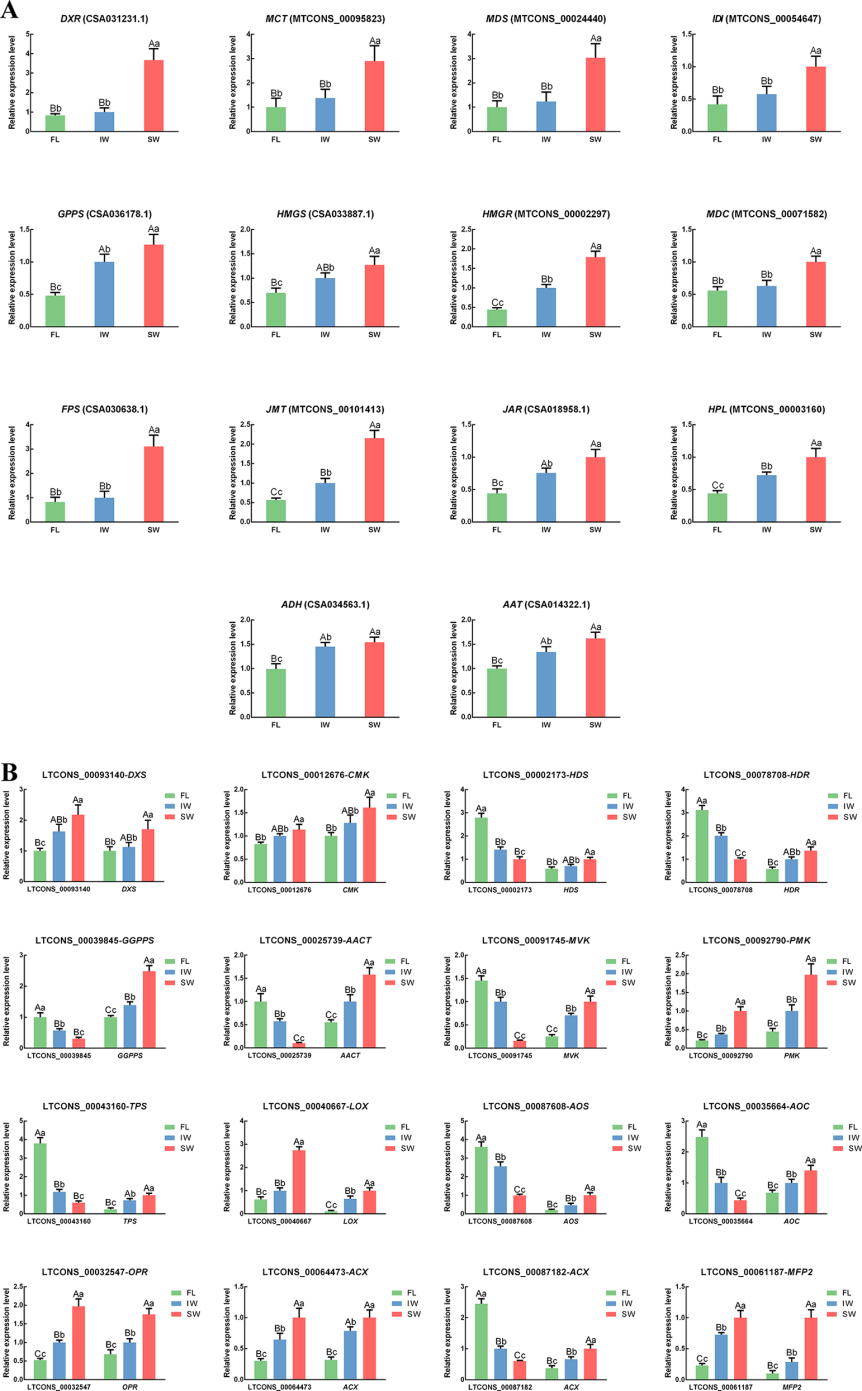
The expression patterns of **(A)** differentially expressed genes (DEGs); **(B)** differentially expressed long non-coding RNAs (DE-lncRNAs) and their target genes in jasmonic acid/methyl jasmonate (JA/MeJA) biosynthesis and signal transduction pathway and terpenoid metabolic pathway determined by qRT-PCR. Data are presented as mean ± standard deviation (SD). Lowercase letter indicates significant difference (*p* < 0.05); uppercase letter indicates highly significant difference (*p* < 0.01).

To verify the accuracy of the lncRNA and target gene expression patterns, we conducted qRT-PCR analyses of 18 structural genes involved in the terpenoid metabolic pathway. The transcript levels of eight structural genes involved in JA/MeJA biosynthesis and three structural genes involved in the HPL pathway were also analyzed by qRT-PCR. We also conducted qRT-PCR analyses to determine the expression levels of the following lncRNAs involved in the terpenoid metabolic pathway and the JA/MeJA biosynthesis and signal transduction pathway: LTCONS_00093140 (targeting *DXS*), LTCONS_00012676 (targeting *CMK*), LTCONS_00002173 (targeting *HDS*), LTCONS_00078708 (targeting *HDR*), LTCONS_00039845 (targeting *GGPPS*), LTCONS_00025739 (targeting *AACT*), LTCONS_00091745 (targeting *MVK*), LTCONS_00092790 (targeting *PMK*), LTCONS_00043160 (targeting *TPS*), LTCONS_00040667 (targeting *LOX*), LTCONS_00087608 (targeting *AOS*), LTCONS_00035664 (targeting *AOC*), LTCONS_00032547 (targeting *OPR*), LTCONS_00064473 (targeting *ACX*), LTCONS_00087182 (targeting *ACX*), and LTCONS_00061187 (targeting *MFP2*). The correlations between the expression of the lncRNAs and their target genes were also analyzed (**Supplementary Table S5**). In the scaffolds, the structures of all important *cis*-regulated genes and their lncRNAs involved in terpenoid metabolism and JA/MeJA biosynthesis and signal transduction were plotted (**Supplementary Figure S5**). The expression trends of selected genes and lncRNAs were similar between the qRT-PCR data and the transcriptome dataset (**Figure 7**). These results confirmed the significant differences in the transcript levels of terpenoid metabolism-related lncRNAs and target genes in the FL vs. IW, FL vs. SW, and IW vs. SW comparisons.

### Relationships Between the Long Non-Coding RNAs and MicroRNAs Involved in Flavonoid Metabolism, Terpenoid Metabolism, and Jasmonic Acid/Methyl Jasmonate Biosynthesis and Signal Transduction

To analyze the relationships between the lncRNAs and miRNAs involved in flavonoid metabolism, terpenoid metabolism, and JA/MeJA biosynthesis and signal transduction, the psRNATarget program was used to predict the potential regulatory relationships between lncRNAs and miRNAs. A total of 41 lncRNAs involved in these three metabolic pathways were predicted to be the targets of 76 miRNAs from 35 families. Additionally, 133 lncRNA–miRNA interacting pairs were identified (**Supplementary Table S10**). These lncRNAs included 28 that were targeted by multiple miRNAs and 13 that were regulated by only one miRNA. Similarly, we identified 36 miRNAs that targeted only one lncRNA and 40 miRNAs with more than two target lncRNAs. Thus, most of the identified lncRNAs were regulated by multiple miRNAs.

**Figure 8:**
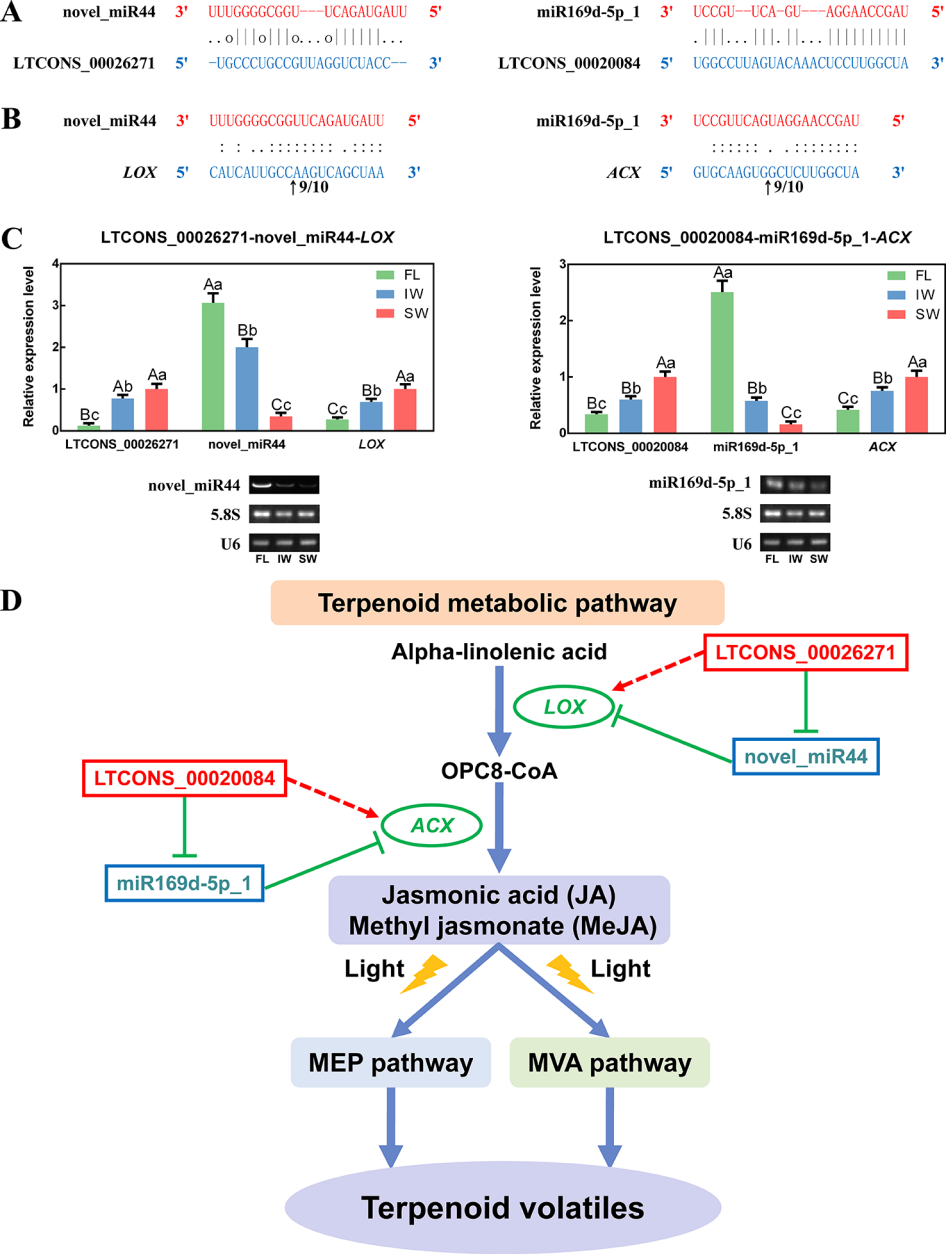
The long non-coding RNAs (lncRNAs) acting as endogenous target mimics (eTMs) of microRNAs (miRNAs), qRT-PCR validation of lncRNAs, miRNAs, and messenger RNAs (mRNAs), and the eTM regulatory network. **(A)** Two lncRNAs were identified as eTMs of related miRNAs. **(B)** The mRNA cleavage sites of miRNAs identified by 5′ RNA ligase-mediated rapid amplification of cDNA ends (5′ RLM-RACE). The arrows indicate the 5′ termini of mRNA fragments, as identified by the RLM-RACE product, with the frequency of clones shown above. The numbers indicate the fraction of cloned PCR products terminating at different positions. **(C)** qRT-PCR validation of novel_miR44 with its target gene (*LOX*) and eTM (LTCONS_00026271), and miR169d-5p_1 with its target gene (*ACX*) and eTM (LTCONS_00020084). Below the histograms are the gel electrophoretograms of miRNAs, *5.8S rRNA*, and *U6 snRNA*. Data are presented as mean ± standard deviation (SD). Lowercase letter indicates significant difference (*p* < 0.05); uppercase letter indicates highly significant difference (*p* < 0.01). **(D)** The eTM regulatory network involved in jasmonic acid/methyl jasmonate (JA/MeJA) biosynthesis and signal transduction pathway. The solid line with the arrow represents direct regulation; the dashed line with the arrow represents indirect regulation; the T-shaped line represents negative regulation.

As novel regulatory factors, eTMs inhibit miRNA functions by binding to miRNAs, which indirectly affects the expression of miRNA targets. Some lncRNAs function as eTMs, thereby contributing to the regulation of miRNAs. In the present study, two lncRNAs detected in the transcriptome dataset were predicted to be potential eTMs (LTCONS_00026271 and LTCONS_00020084) for two miRNAs (novel_miR44 and miR169d-5p_1) associated with the JA/MeJA biosynthesis and signal transduction pathway, respectively (**Figure 8A**). Moreover, we determined that novel_miR44 targets *LOX*, whereas miR169d-5p_1 targets *ACX* (**Supplementary Table S11**). To identify the cleavage sites, the 5′ RLM-RACE PCR products for *LOX* and *ACX* were analyzed. The sequencing results were shown in **Supplementary Figure S8**. The results indicated that *LOX* can be regulated by cleavage in the binding region between the 10^th^ and 11^th^ bases from the 5′ end pairing of novel_miR44, and *ACX* can be regulated by cleavage in the binding region between the 9^th^ and 10^th^ bases from the 5′ end pairing of miR169d-5p_1 (**Figure 8B**). This proved that *LOX* and *ACX* are directly cleaved by miRNAs.

To further explore the regulatory networks of eTMs with miRNAs and target genes, the expression patterns of selected eTMs (LTCONS_00026271 and LTCONS_00020084), miRNAs (novel_miR44 and miR169d-5p_1), and target genes (*LOX*: MTCONS_00093153 and *ACX*: MTCONS_00008303) were analyzed. According to qRT-PCR analyses (**Figure 8C**), the expression levels of LTCONS_00026271 and *LOX* were significantly higher in the SW than in the FL and IW, whereas the expression level of novel_miR44 was lower in the SW than in the FL and IW. Additionally, LTCONS_00020084 and *ACX* expression levels were also higher in the SW than in the FL and IW, whereas miR169d-5p_1 expression exhibited the opposite pattern. Moreover, the expression profiles of novel_miR44 and miR169d-5p_1 were confirmed by RT-PCR. These profiles were consistent with the miRNA expression levels determined by qRT-PCR. To further validate the correlations among expression levels, the expression of lncRNAs, miRNAs, and mRNAs in diverse leaf samples (bud, first leaf, second leaf, and third leaf) was investigated by qRT-PCR (**Supplementary Figure S9**). On the basis of these results, we detected negative relationships between eTMs and miRNAs, as well as between miRNAs and target genes. Conversely, the expression levels of eTMs and target genes were positively correlated. The lncRNA–miRNA–mRNA regulatory network in the JA/MeJA biosynthesis and signal transduction pathway was also constructed (**Figure 8D**). Together, these results suggested that lncRNAs may affect the expression of related mRNAs through miRNAs.

## Discussion

### The Expression Levels of the Identified Differentially Expressed Long Non-Coding RNAs and Their Target Genes Involved in Flavonoid Metabolism Were Associated With the Low Total Flavonoid and Catechin Contents in the Solar-Withered Leaves

Previous studies (Chen et al., 2011a) proved that tea flavor is greatly influenced by the manufacturing process. Withering is the first indispensable process for improving oolong tea flavors because of the associated changes to the contents of some tea flavor-related compounds. Among these flavor-related compounds, flavonoids and catechins are the representative factors for evaluating oolong tea flavor quality (Balentine et al., 1997; Yang et al., 2013). However, the total polyphenol, flavonoid, and catechin contents in fresh and withered oolong tea leaves had not been determined. Thus, we analyzed the total polyphenol, flavonoid, and catechin contents as well as the abundance of eight individual catechins in the FL, IW, and SW.

In this study, the SW had lower levels of total polyphenols, total flavonoids, total catechins, and individual catechins in the FL vs. SW and IW vs. SW comparisons. In contrast, the lignin content was significantly higher in the SW than in the FL and IW. Additionally, five individual catechins (EGC, CG, GCG, ECG, and EGCG) were all less abundant in the SW than in the FL and IW. However, the molecular mechanisms underlying the differences in the total polyphenol, total flavonoid, and catechin contents in the SW and IW remain unclear. Moreover, previous studies concluded that lncRNAs play important regulatory roles at the transcript level (Liu et al., 2018b; Lin et al., 2019; Varshney et al., 2019). However, there has been relatively little research on the lncRNAs in tea plants, with no published reports regarding the regulatory mechanisms of lncRNAs related to the processing of tea leaves. Thus, identifying and characterizing the lncRNAs may be useful for clarifying the solar-withering process.

To explore the regulatory mechanisms of lncRNAs during the withering process, we identified the KEGG pathways enriched among the target genes of DE-lncRNAs. The KEGG pathway analyses revealed that the flavonoid metabolic pathway is one of the representative pathways among the FL, IW, and SW. Lignin metabolism and flavonoid metabolism are parallel pathways that compete for the same substrates. Regarding the lncRNA regulatory activities in the FL, IW, and SW, 10 regulatory relationships involving lncRNAs and mRNAs related to flavonoid and lignin metabolism were analyzed by qRT-PCR, including seven lncRNA–mRNA regulatory pairs in which the lncRNA positively regulated the target genes as well as three regulatory pairs in which the lncRNA negatively regulated the target genes.

Compared with the expression levels in the IW, the down-regulated expression of lncRNAs (LTCONS_00054003 and LTCONS_00060939) in the SW inhibited the normal expression of their target genes (*4CL* and *CHI*). Moreover, five up-regulated lncRNAs (LTCONS_00031811, LTCONS_00030131, LTCONS_00101116, LTCONS_00001863, and LTCONS_00090121) in the SW up-regulated the expression of their target genes (*FLS*, *HCT*, *CCR*, and *CAD*). Moreover, some lncRNAs can negatively regulate the expression of their target genes (Wang et al., 2014; Chekanova, 2015; Liu et al., 2015). In the current study, three pairs of negatively regulated lncRNA–mRNA relationships were detected for the flavonoid metabolic pathway. High expression levels of LTCONS_00056216 and LTCONS_00044497 suppressed the expression of *F3H*, *F3’H*, and *CAD* in the SW, whereas low LTCONS_00000233 expression in the SW up-regulated *CAD* expression.

Among the flavonoid metabolism-related genes, *4CL* is a key gene for catalyzing reactions involving coumarate and its derivatives, thereby generating substrates for the subsequent synthesis of flavonoids and other substances (Ehlting et al., 1999; Yun et al., 2005). Many studies confirmed that high *4CL* expression levels promote flavonoid biosynthesis (Sun et al., 2013b; Gao et al., 2015), whereas down-regulated *4CL* expression decreases flavonoid accumulation (Zhou et al., 2015). In the current study, the low expression of LTCONS_00054003 inhibited *4CL* expression in the SW. Similarly, the *4CL* expression levels in *Populus tomentosa* (Chen et al., 2015) and *Paulownia tomentosa* (Cao et al., 2018) are also regulated by related lncRNAs. Moreover, a previous study proved that *4CL* has important effects on the production of catechins in tea leaves (Rani et al., 2009). Thus, the *4CL* expression level is positively correlated with the total flavonoid and catechin contents of tea leaves. Additionally, low LTCONS_00054003 and *4CL* expression levels may not be conducive to the accumulation of these compounds in the SW. In the flavonoid metabolic pathway, *CHI*, which is expressed downstream of *4CL*, is mainly involved in catalyzing the intramolecular cyclization of chalcone to form naringenin in plants (Jez et al., 2000; Gensheimer and Mushegian, 2004). In various grapevine tissues, the *CHI* expression pattern is positively correlated with the total flavonoid content (Wang et al., 2019d). Additionally, withering reportedly decreases the expression of *CHI* and the accumulation of catechins, including EGCG, in tea leaves (Wang et al., 2019b). Consistent with these findings, we observed that the *CHI* transcript level and the EGCG content were lower in the SW than in the IW. An earlier investigation indicated that some lncRNAs help regulate the transcription of *CHI* (Varshney et al., 2019). We determined that the LTCONS_00060939 and *CHI* expression levels were lower in the SW than in the IW, implying that a high LTCONS_00060939 expression level is important for inhibiting *CHI* expression.

During the solar-withering process, the withered leaves are exposed to diverse stresses, including drought, heat, and UV irradiation, whereas the leaves withered indoors are mainly affected by drought stress (Cho et al., 2007; Gui et al., 2015). Solar-withering accelerates the dehydration of tea leaves, with a more severe drought stress than that experienced by leaves withered indoors. Accordingly, we propose that solar-withering helps to decrease the *CHI* expression level and the EGCG content. The *F3H* and *F3’H* genes also influence flavonoid metabolism. Previous studies revealed that the catechin content of tea plants are positively correlated with the *F3H* and *F3’H* transcript levels (Zhou et al., 2016; Guo et al., 2019b). In the current study, we observed a negative correlation between the expression levels of lncRNAs and these two genes. Therefore, the up-regulated expression of LTCONS_00056216 and LTCONS_00044497 in the SW likely inhibited the expression of *F3H* and *F3’H*, which is unfavorable for the accumulation of flavonoids and catechins in the SW.

Expressed downstream of the flavonoid metabolic pathway, *FLS* is responsible for converting dihydroflavonols to flavonols. It competes with DFR for the substrate dihydroflavonols. Because DFR promotes catechin biosynthesis, FLS has an inhibitory effect on catechin production. A previous study indicated that *FLS* expression is negatively correlated with the biosynthesis of catechins, especially EGCG (Xiong et al., 2013). Thus, we speculate that the low *FLS* expression levels in the IW had little effect on catechin biosynthesis, whereas the high *FLS* expression levels in the SW inhibited catechin accumulation. Additionally, high lncRNA (LTCONS_00031811) expression levels positively regulate *FLS* expression, which may explain the high *FLS* expression levels detected in the SW. Three other genes (*CCR*, *CAD*, and *HCT*) belong to the lignin biosynthesis pathway, and their expression levels directly affect the lignin accumulation in plants (Vanholme et al., 2010). Regarding phenylpropanoid metabolism, lignin biosynthesis and flavonoid metabolism represent parallel pathways. Many studies revealed that the lignin content is positively correlated with the expression of *CCR*, *CAD*, and *HCT* (Vanholme et al., 2008; Thévenin et al., 2011; Wang et al., 2019e). Similar to the corresponding *Arabidopsis thaliana* pathways, (Besseau et al., 2007), lignin metabolism and flavonoid metabolism in tea plants may compete for the same precursor substances. Therefore, compared with expression levels in the IW, the up-regulated expression of *CCR*, *CAD*, and *HCT* in the SW may induce lignin biosynthesis and inhibit flavonoid biosynthesis, thereby decreasing the abundance of the precursor substance for catechin biosynthesis in the SW. These changes may help to explain why total polyphenol, total flavonoid, and catechin contents may be high in the IW, but not in the SW.

The above-mentioned results suggest that high *FLS*, *CCR*, *CAD*, and *HCT* transcript levels and seven up-regulated lncRNAs (LTCONS_00056216, LTCONS_00044497, LTCONS_00031811, LTCONS_00001863, LTCONS_00090121, LTCONS_00030131, and LTCONS_00101116) as well as low *4CL*, *CHI*, *F3H*, and *F3’H* transcript levels and three down-regulated lncRNAs (LTCONS_00054003, LTCONS_00060939, and LTCONS_00000233) in the SW are closely associated with the low abundance of total polyphenols, flavonoids, and catechins in the SW. Thus, we propose that because of the regulation of related lncRNAs, the expression of genes involved in the flavonoid biosynthetic pathway is inhibited, whereas the expression of genes related to the lignin biosynthetic pathway is up-regulated, thereby decreasing the amounts of total flavonoids and catechins, especially the galloylated catechins, in the SW. Decreased galloylated catechin, polyphenol, and flavonoid contents may help to reduce the bitterness and astringency of tea leaves and moderately improve the palatability of tea infusions. Therefore, solar-withering is conducive to the production of weakly astringent oolong tea leaves with a mellow taste.

### The Expression Levels of the Identified Differentially Expressed Long Non-Coding RNAs and Their Target Genes Involved in Terpenoid Metabolism Are Related to the Considerable Abundance of Terpenoid Volatiles in the Solar-Withered Leaves

Terpenoids are one of the important components in oolong tea, and their content is positively correlated with the aroma quality of oolong tea (Chen et al., 2011a). Some gene families involved in the terpenoid metabolic pathway have expanded in the tea genome, further suggesting that terpenoids are crucial for the aroma of tea leaves (Wei et al., 2018). In the current study, the abundance of terpenoid volatiles, including β-ocimene, limonene, γ-terpinene, α-farnesene, and β-myrcene, was higher in the SW than in the IW. Moreover, we identified nine lncRNA–mRNA regulatory relationships associated with terpenoid metabolism, of which three were positive relationships and six were negative relationships. Our qRT-PCR results confirmed that LTCONS_00093140, LTCONS_00012676, and LTCONS_00092790 expression levels were higher in the SW than in the IW. Additionally, their target genes (*DXS*, *CMK*, and *PMK*) were more highly expressed in the SW than in the IW. The expression levels of six other lncRNAs (LTCONS_00002173, LTCONS_00078708, LTCONS_00039845, LTCONS_00025739, LTCONS_00091745, and LTCONS_00043160) were lower in the SW than in the IW, whereas their target genes were more highly expressed in the SW than in the IW. Therefore, these lncRNAs have a negative regulatory effect on the expression of their target genes (*HDS*, *HDR*, *GGPPS*, *AACT*, *MVK*, and *TPS*).

Among the genes related to terpenoid metabolism, *DXS*, *CMK*, *HDS*, and *HDR* belong to the methylerythritol phosphate (MEP) pathway, whereas *AACT*, *MVK*, and *PMK* belong to the mevalonate pathway (Hu et al., 2018; Wang et al., 2019b). In the mevalonate pathway, acetyl-CoA acts as a substrate. In contrast, the metabolic substrates in the MEP pathway are pyruvate and glyceraldehyde-3-phosphate. However, the final products of both of these pathways are isopentenyl diphosphates (IPPs). Moreover, IPP biosynthesis will directly affect the yield of downstream terpenoids (Vranová et al., 2013). Additionally, several studies confirmed that the expression levels of the genes involved in the MEP pathway are positively correlated with the terpenoid yield (Ma et al., 2012; Webb et al., 2013; Han et al., 2016a). Consistent with these earlier findings, an analysis of the transcription of the target genes related to terpenoid metabolism revealed that the higher expression of seven upstream genes (*DXS*, *CMK*, *HDS*, *HDR*, *AACT*, *MVK*, and *PMK*) in the SW than in the IW was regulated by lncRNAs. Thus, the high terpenoid metabolite contents (e.g., γ-terpinene, α-farnesene, and β-myrcene) in the SW may be due to the transcription of these lncRNAs and their target genes related to terpenoid metabolism, which enhances the metabolic flux toward terpenoid biosynthesis and their precursors, especially IPP. The remaining two target genes, *GGPPS* and *TPS*, are expressed downstream of terpenoid metabolism. Previous studies indicated that *GGPPS* is important for catalyzing the biosynthesis of carotenoids from geranylgeranyl diphosphate (Okada et al., 2000; Song et al., 2017). Carotenoids can be further degraded to aromatic compounds, such as β-ionone, which influences tea flavors because of its low olfactory threshold (Lewinsohn et al., 2005; Schuh and Schieberle, 2006; Dudareva et al., 2013). In our study, the β-ionone content was higher in the SW than in the IW. After being negatively regulated by LTCONS_00039845, the *GGPPS* transcript level was also higher in the SW than in the IW. Therefore, the *GGPPS* expression level is positively correlated with the β-ionone content, whereas the LTCONS_00039845 expression level negatively regulates the abundance of β-ionone. Accordingly, high *GGPPS* expression levels may be crucial for β-ionone biosynthesis. Additionally, sunlight and UV treatments are conducive for the accumulation of β-ionone in plants (Bouvier et al., 2005; Jang et al., 2010; Song et al., 2015). We propose that the observed greater abundance of β-ionone in the SW than in the IW was in part due to the effects of solar-withering. Because β-ionone is one of the characteristic aroma components of oolong tea, its higher content in the SW than in the IW implies that solar-withering is more conducive to the formation of a high-quality aroma in oolong tea than indoor-withering. The *TPS* gene is indispensable for the production of the most abundant and structurally diverse terpenoids influencing tea aroma. Moreover, high *TPS* expression level facilitates the conversion of precursors to various terpenoids (Chen et al., 2011b; Zhao et al., 2018). In the present study, high LTCONS_00043160 expression level inhibited *TPS* expression in the IW. However, LTCONS_00043160 was expressed at a low level in the SW, with minimal effects on *TPS* expression. Therefore, *TPS* was expressed more highly in the SW than in the IW. Furthermore, the abundance of terpenoid volatiles was higher in the SW than in the IW, suggesting the terpenoid volatile contents are affected by the expression of *TPS* and its related lncRNA.

Relative to the corresponding expression levels in the IW, the SW had nine up-regulated genes (*DXS*, *CMK*, *HDS*, *HDR*, *AACT*, *MVK*, *PMK*, *GGPPS*, and *TPS*), three up-regulated lncRNAs (LTCONS_00093140, LTCONS_00012676, and LTCONS_00092790), and six down-regulated lncRNAs (LTCONS_00002173, LTCONS_00078708, LTCONS_00039845, LTCONS_00025739, LTCONS_00091745, and LTCONS_00043160) related to terpenoid metabolism. Plant lncRNAs are increasingly being identified as factors regulating secondary metabolism (Li et al., 2017; Wong and Matus, 2017; Wang et al., 2018b; Chen et al.,2018 c). Consistent with previous research, we observed that during the withering process, terpenoid metabolism was affected by the expression of related genes as well as lncRNAs. This suggests the synergism between lncRNAs and their target genes is related to terpenoid metabolism during the withering of oolong tea leaves. After the solar-withering treatment, oolong tea products have a floral and fruity aroma. However, indoor-withering results in oolong tea products with a dull aroma (Kobayashi et al., 1985). Combined with the expression of lncRNAs and their target genes related to terpenoid metabolism, high terpenoid contents may promote the development of a higher quality aroma in the SW than in the IW. Moreover, the β-ionone content was higher in the SW than in the IW, whereas the (*Z*)-3-hexenal and (*E*)-2-hexenal contents exhibited the opposite pattern. The expression of the *ADH* gene reportedly induces the degradation of hexenal (Hu et al., 2018). Therefore, the decrease in the hexenal content of the SW may be related to the significant increase in *ADH* expression. In contrast to the effects of β-ionone on oolong tea aroma, (*Z*)-3-hexenal and (*E*)-2-hexenal are the most important tea components that produce a grassy aroma, which is undesirable for tea (Zheng et al., 2016). Therefore, the solar-withering process results in high terpenoid and β-ionone levels and low (*Z*)-3-hexenal and (*E*)-2-hexenal levels in the SW, resulting in a slight grassy aroma, but a stronger floral and fruity aroma. This helps to explain why solar-withering is better than indoor-withering regarding the production of a higher quality oolong tea aroma.

### High Jasmonic Acid and Methyl Jasmonate Contents and an Endogenous Target Mimic-Related Regulatory Mechanism Promote the Accumulation of Terpenoids in Solar-Withered Leaves

Light is a major environmental factor affecting the accumulation of terpenoid metabolites as well as the expression patterns of numerous genes involved in the terpenoid metabolic pathway (Chen et al., 2004; Rodríguez-Concepción, 2006; Cordoba et al., 2009). Previous studies confirmed the positive effects of light on the transcription of *DXS*, *CMK*, *HDS*, and *HDR* (Hsieh and Goodman, 2005; Nakamura et al., 2007; Huang et al., 2009; Xu et al., 2018). In the present study, the *DXS*, *CMK*, *HDS*, and *HDR* transcript levels were significantly higher in the SW than in the IW, implying that during the withering of tea leaves, sunlight induces the expression of these genes better than artificial indoor lights. A recent study detected many light-specific *cis*-acting elements in the promoter region of most genes in the MEP pathway (Xu et al., 2018). An exposure to sunlight during solar-withering activates the expression of *DXS*, *CMK*, *HDS*, and *HDR* and the accumulation of terpenoids. The light intensity of solar-withering is stronger than that of indoor-withering, which promotes the up-regulated expression of the genes related to the MEP pathway and enhances terpenoid metabolism, thereby increasing the abundance of terpenoid volatiles in the SW. Therefore, the MEP pathway during the withering process is stimulated by light. In *A. thaliana* seedlings, light can also significantly induce the expression of *AtGGPPS11* downstream of terpenoid metabolism (Vranová et al., 2013). In tea plants, *TPS* expression is regulated by light (Fu et al., 2015). Consistent with the results of previous research, we observed that *GGPPS* and *TPS* transcript levels were higher in the SW than in the IW. Moreover, β-ionone and terpenoid contents were highly associated with the *GGPPS* and *TPS* expression levels, respectively. The lack of sufficient light during the indoor-withering process contributed to the lower expression levels of the terpenoid metabolism-related genes as well as the decreased accumulation of terpenoid volatiles in the IW than in the SW. Thus, solar-withering induces the up-regulated expression of *DXS*, *CMK*, *HDS*, *HDR*, *GGPPS*, and *TPS* and increases the accumulation of β-ionone and the terpenoid volatiles in the SW, ultimately resulting in oolong tea leaves with a floral and fruity aroma.

Phytohormones are important bioactive molecules that function as signaling molecules during the regulation of the synthesis of volatiles (Di et al., 2019; Li et al., 2019). Earlier studies indicated that jasmonate enhances the formation of terpenoid volatiles (Ament et al., 2004; Garde-Cerdán et al., 2018; Wasternack and Strnad, 2019). However, little is known about the specific role of jasmonate during the processing of tea leaves. To investigate the effects of JA and MeJA on the tea aroma during the withering process, we analyzed the JA and MeJA contents in the FL, IW, and SW. The abundance of both phytohormones was higher in the SW than in the IW and FL. Moreover, seven lncRNA–mRNA regulatory relationships associated with the JA/MeJA biosynthesis and signal transduction pathway were identified. Four of these regulatory relationships were positive, whereas the other three were negative. Recent studies confirmed that JA/MeJA biosynthesis involves a series of genes, including *LOX*, *AOS*, *AOC*, *OPR*, *ACX*, and *MFP2* (Wasternack and Feussner, 2018; Zhu et al., 2018). Among these genes, *LOX* mediates the first step of JA/MeJA biosynthesis. Consequently, the up-regulated *LOX* expression in plants in response to various stresses will induce JA/MeJA accumulation (Chen et al., 2016). In the current study, a high LTCONS_00040667 level promoted *LOX* expression in the SW. Additionally, JA and MeJA levels were also high in the SW. These observations imply that the LTCONS_00040667 and *LOX* transcription levels are highly associated with JA and MeJA contents. Additionally, high LTCONS_00040667 and *LOX* expression levels are conducive to JA/MeJA biosynthesis in the SW.

In the AOS pathway, five genes (*AOS*, *AOC*, *OPR*, *ACX*, and *MFP2*) have key roles related to JA/MeJA biosynthesis. High expression levels of *AOS*, *AOC*, *OPR*, *ACX*, and *MFP2* lead to the accumulation of JA and MeJA (Wasternack and Hause, 2013; Mehta et al., 2017; Wasternack and Feussner, 2018; Islam et al., 2019). Moreover, the up-regulated expression of *JMT* increases the MeJA content (Shi et al., 2015). During the tea-withering process, high LTCONS_00032547, LTCONS_00064473, and LTCONS_00061187 expression levels up-regulated the expression of *OPR*, *ACX*, and *MFP2* in the SW. Three other lncRNAs (LTCONS_00087608, LTCONS_00035664, and LTCONS_00087182), which were expressed at low levels in the SW, negatively regulate the expression of their target genes (*AOS*, *AOC*, and *ACX*). Consequently, these target genes were more highly expressed in the SW than in the IW. Therefore, these lncRNAs appear to up-regulate the expression of JA/MeJA-related genes in the SW *via* multiple regulatory mechanisms. Previous studies revealed that the jasmonate biosynthesis pathway in plants may be induced under abiotic stress conditions, and many jasmonate-related metabolites are important for plant defenses and plant–environment interactions (Kazan, 2015; Per et al., 2018). Similarly, leaves that undergo solar-withering are exposed to more diverse stresses, including drought, heat, and UV radiation, than leaves that are withered indoors, which are primarily affected by drought stress. Therefore, the degree of abiotic stress is greater for the SW than for the IW. Additionally, the accumulation of jasmonate in plants increases following the expression of genes related to the JA/MeJA biosynthetic pathway in response to biotic and abiotic stresses (Zhang et al., 2019; Zhou et al., 2019b). In the present study, the expression of JA/MeJA biosynthesis-related genes was positively correlated with the JA and MeJA contents. The observed up-regulated expression of the JA/MeJA biosynthesis-related genes in the SW promotes the accumulation of JA and MeJA, which activates the downstream terpenoid metabolic pathway and increases the synthesis of related terpenoid volatiles.

A previous study proved that some lncRNAs can act as eTMs that bind to specific miRNAs, thereby preventing the miRNAs from cleaving the target transcripts, which represents a new mechanism by which lncRNAs regulate miRNAs in plants (Wu et al., 2013). However, in tea plants, lncRNAs functioning as eTMs have not been identified and the regulation of miRNAs and mRNAs by eTMs has not been established. An analysis of the transcriptome dataset in the current study revealed two lncRNAs (LTCONS_00026271 and LTCONS_00020084) that may serve as eTMs for two miRNAs (novel_miR44 and miR169d-5p_1) involved in the JA/MeJA biosynthesis and signal transduction pathway. The subsequent analysis of the genes targeted by these two miRNAs indicated that novel_miR44 targets *LOX*, whereas miR169d-5p_1 targets *ACX*. Compared with corresponding levels in the IW, the LTCONS_00026271 and *LOX* expression levels were higher in the SW, whereas the novel_miR44 expression level was lower. Similarly, the miR169d-5p_1 expression level was significantly lower in the SW than in the IW, but the LTCONS_00020084 and *ACX* expression levels were consistently higher in the SW than in the IW. Previous studies indicated that high lncRNA expression levels lead to the binding of miRNAs, thereby protecting the target transcripts from being cleaved (Franco-Zorrilla et al., 2007; Meng et al., 2012). During the tea-withering process, high LTCONS_00026271 and LTCONS_00020084 transcript levels result in lncRNAs that serve as eTMs to block the activities of specific miRNAs to promote the expression of *LOX* and *ACX*. The increased *LOX* and *ACX* help induce the accumulation of JA. Furthermore, the terpenoid metabolic pathway is activated *via* JA/MeJA signal transduction to promote the production of terpenoid volatiles in the SW. Thus, the eTM-related regulatory mechanism (LTCONS_00026271-novel_miR44-*LOX* and LTCONS_00020084-miR169d-5p_1-*ACX*) in the SW is crucial for increasing terpenoid contents.

Sunlight, high JA and MeJA contents, and the eTM-related regulatory mechanism promote terpenoid metabolism during tea withering. It is possible that sunlight and high JA and MeJA contents have a cumulative effect on terpenoid production. The JA and MeJA contents were higher in the IW than in the FL. Thus, the expression of the genes and the abundance of the metabolites related to terpenoid metabolism were higher in the IW than in the FL. Compared with the IW, the SW, which was exposed to a greater light intensity, had higher JA and MeJA contents. Therefore, the transcript levels of the terpenoid metabolism-related genes and the abundance of terpenoid metabolites were greater in the SW than in the IW. Additionally, the eTM-related regulatory mechanism in the SW was crucial for increasing the JA, MeJA, and terpenoid contents. Therefore, sunlight, high JA and MeJA contents, and eTMs in the SW contribute to the accumulation of aroma-related terpenoids. These findings also confirm that solar-withering is better than indoor-withering regarding the production of oolong tea with a high-quality aroma.

## Conclusions

**Figure 9:**
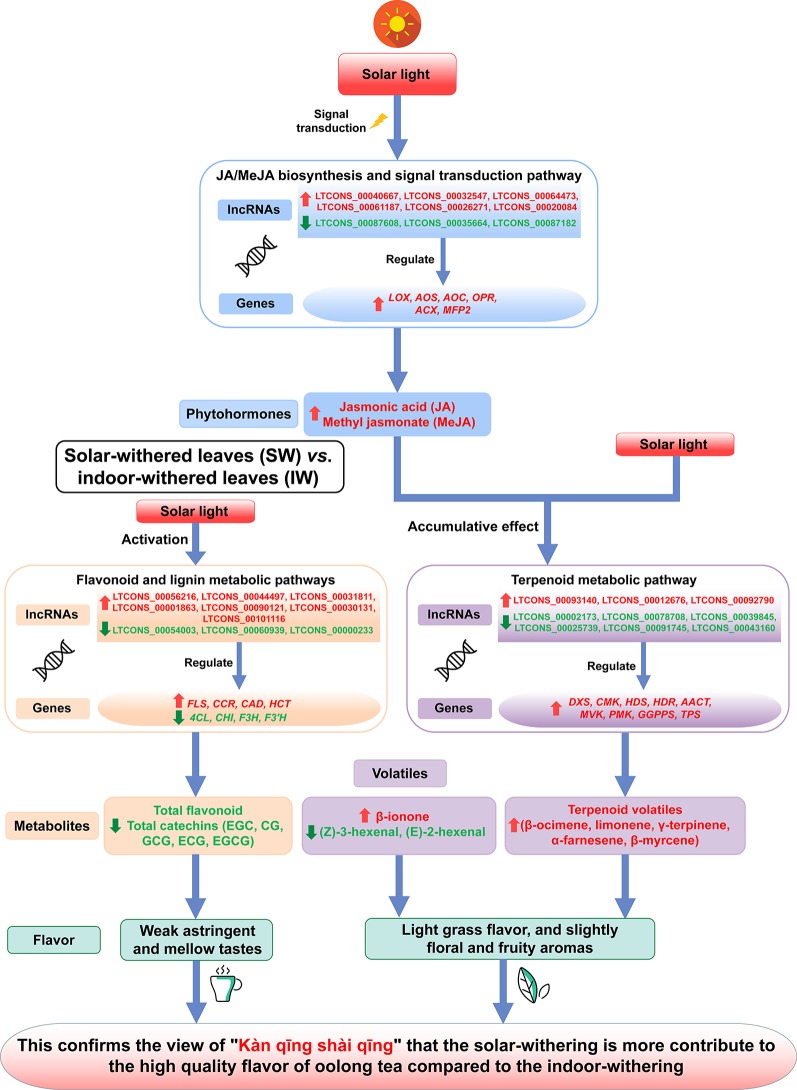
The regulatory network of key long non-coding RNAs (lncRNAs), genes, and metabolites involved in flavonoid metabolic pathway, terpenoid metabolic pathway, and jasmonic acid/methyl jasmonate (JA/MeJA) biosynthesis and signal transduction pathway.

On the basis of transcriptome and phytochemical analyses, we revealed that the total polyphenol, flavonoid, catechin (EGC, CG, GCG, ECG, and EGCG), (*Z*)-3-hexenal, and (*E*)-2-hexenal contents are lower in the SW than in the IW and FL. In contrast, terpenoid volatiles and β-ionone are more abundant in the SW than in the IW and FL. We also analyzed the expression profiles of DE-lncRNAs and their target genes involved in flavonoid metabolism, terpenoid metabolism, and JA/MeJA biosynthesis and signal transduction in the FL, IW, and SW. The results suggested that the expression of DE-lncRNAs and their target genes involved in the three pathways might be related to the low abundance of total flavonoids and total catechins (EGC, CG, GCG, ECG, and EGCG) and the high levels of terpenoid metabolites in the SW. Additionally, the lncRNA regulatory mechanism plays a key role in the accumulation of related secondary metabolites in the SW. Moreover, sunlight and high JA and MeJA contents may have a cumulative effect on terpenoid levels. Therefore, the terpenoid metabolism-related gene expression levels and the terpenoid metabolite contents are higher in the SW than in the IW. Further analyses of lncRNAs revealed that an eTM-related regulatory mechanism (LTCONS_00026271-novel_miR44-*LOX* and LTCONS_00020084-miR169d-5p_1-*ACX*) in the SW is also a crucial factor for increasing terpenoid contents. In addition to the expression of DE-lncRNAs and their target genes, we revealed that low total flavonoid, catechin (EGC, CG, GCG, ECG, and EGCG), (*Z*)-3-hexenal, and (*E*)-2-hexenal contents as well as high terpenoid volatile and β-ionone levels in the SW contribute to the formation of a weakly astringent and mellow tea flavor, a slight grassy aroma, and a stronger floral and fruity aroma. The results described herein also confirm that the solar-withering treatment is more conducive to the development of a high-quality oolong tea than the indoor-withering treatment (**Figure 9**).

## Data Availability Statement

The datasets generated for this study can be found in the NCBI Sequence Read Archive (accession number PRJNA562623).

## Author Contributions

YG, ZL, and CZhu designed the work; YG, CZhu, and SZ performed the experiments and wrote the paper. YG, CZhu, SZ, HF, XL, and YL analyzed the data. CZhu, SZ, CZho, and LC helped to perform the sequence analysis and revised the paper carefully. All authors have read and approved the manuscript.

## Funding

This work was supported by the Natural Science Foundation of Fujian Province (2018J01701), the Earmarked Fund for China Agriculture Research System (CARS-19), the Special Fund Project for Scientific and Technological Innovation of Fujian Agriculture and Forestry University (CXZX2017164, CXZX2017350, and CXZX2018069), the Rural Revitalization Tea Industry Technical Service Project of Fujian Agriculture and Forestry University (11899170102), the Construction of Plateau Discipline of Fujian Province (102/71201801101), and the Scientific Research Foundation of Horticulture College of Fujian Agriculture and Forestry University (2019B01).

## Conflict of Interest

The authors declare that the research was conducted in the absence of any commercial or financial relationships that could be construed as a potential conflict of interest.
