# Supplementary figures and images for: Transcriptome and Phytochemical Analyses Provide New Insights Into Long Non-Coding RNAs Modulating Characteristic Secondary Metabolites of Oolong Tea (Camellia sinensis) in Solar-Withering
Source: Front Plant Sci. 2019 Dec 27;10:1638. doi: 10.3389/fpls.2019.01638 (PMC6941427; doi:10.3389/fpls.2019.01638)

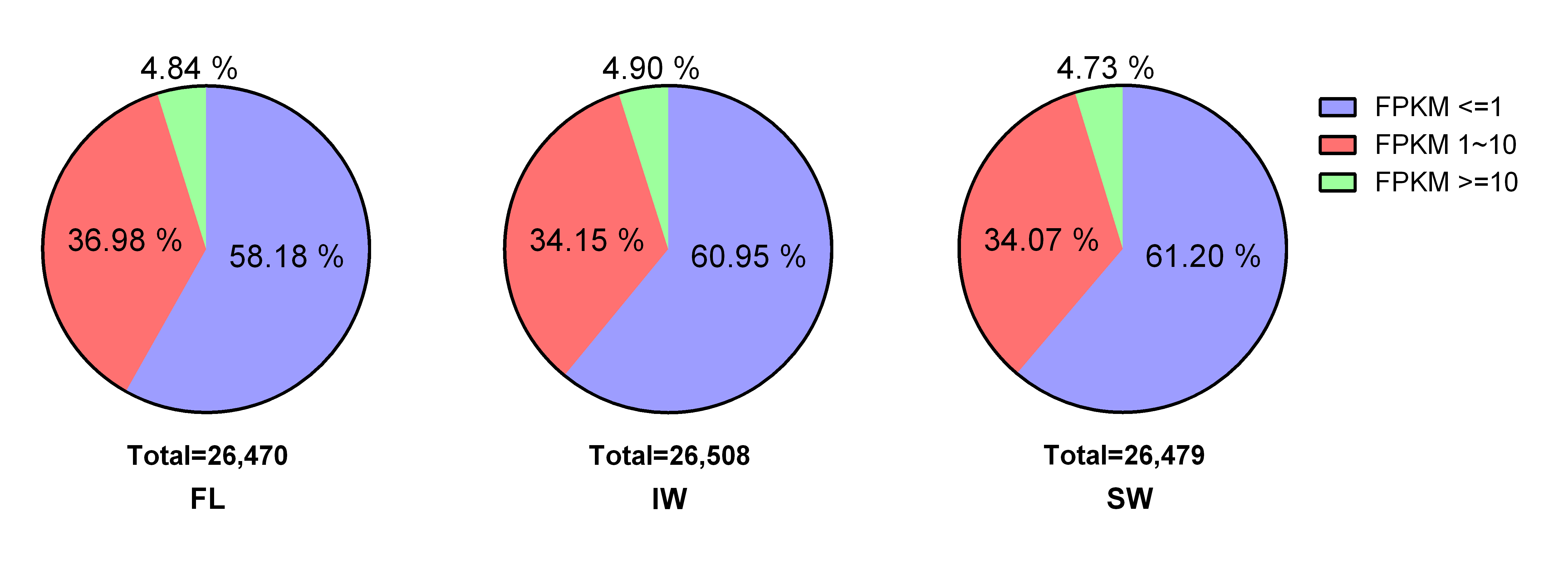

Supplement: Supplementary file 1 [file Image_1.tif]

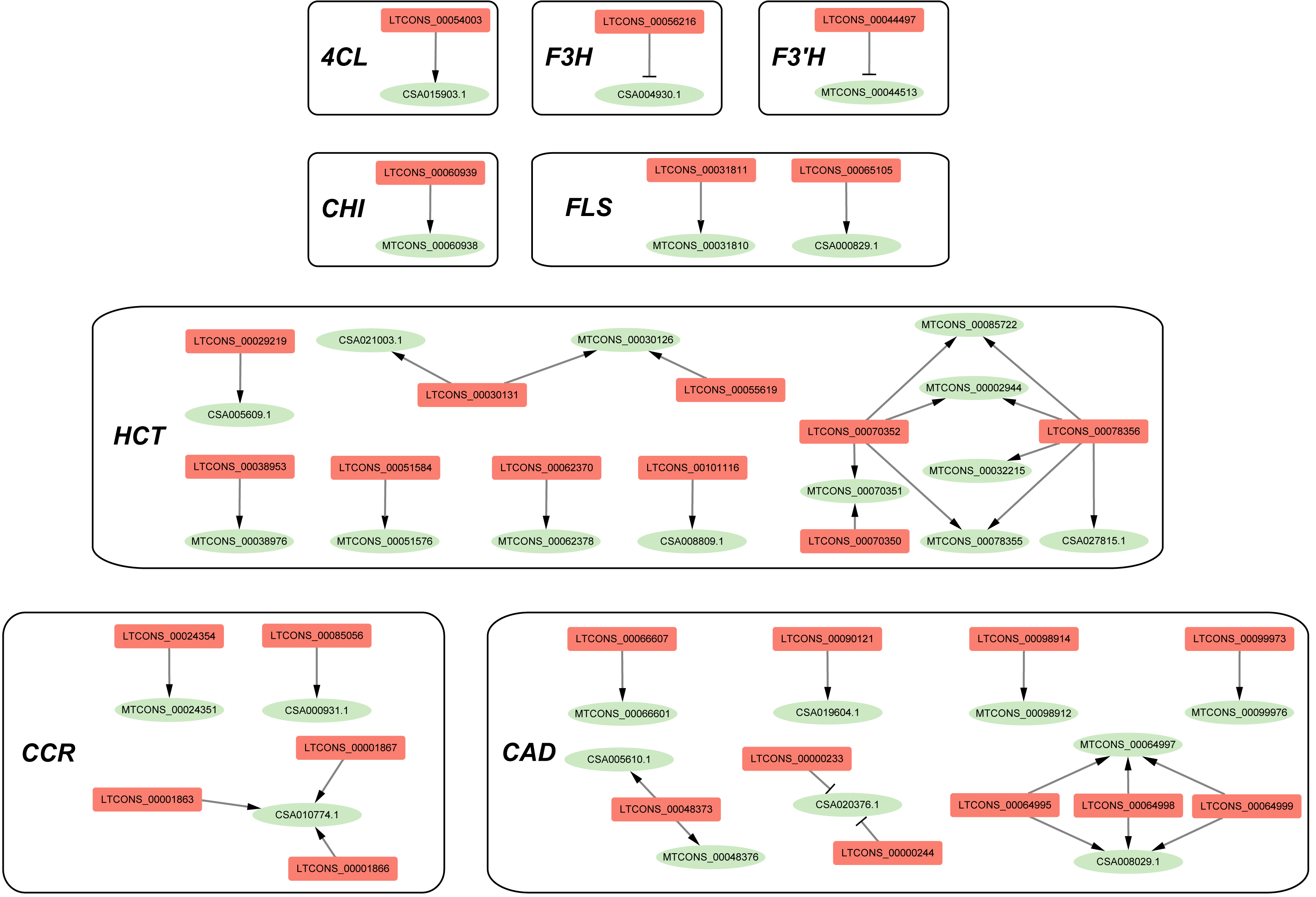

Supplement: Supplementary file 3 [file Image_3.tif]

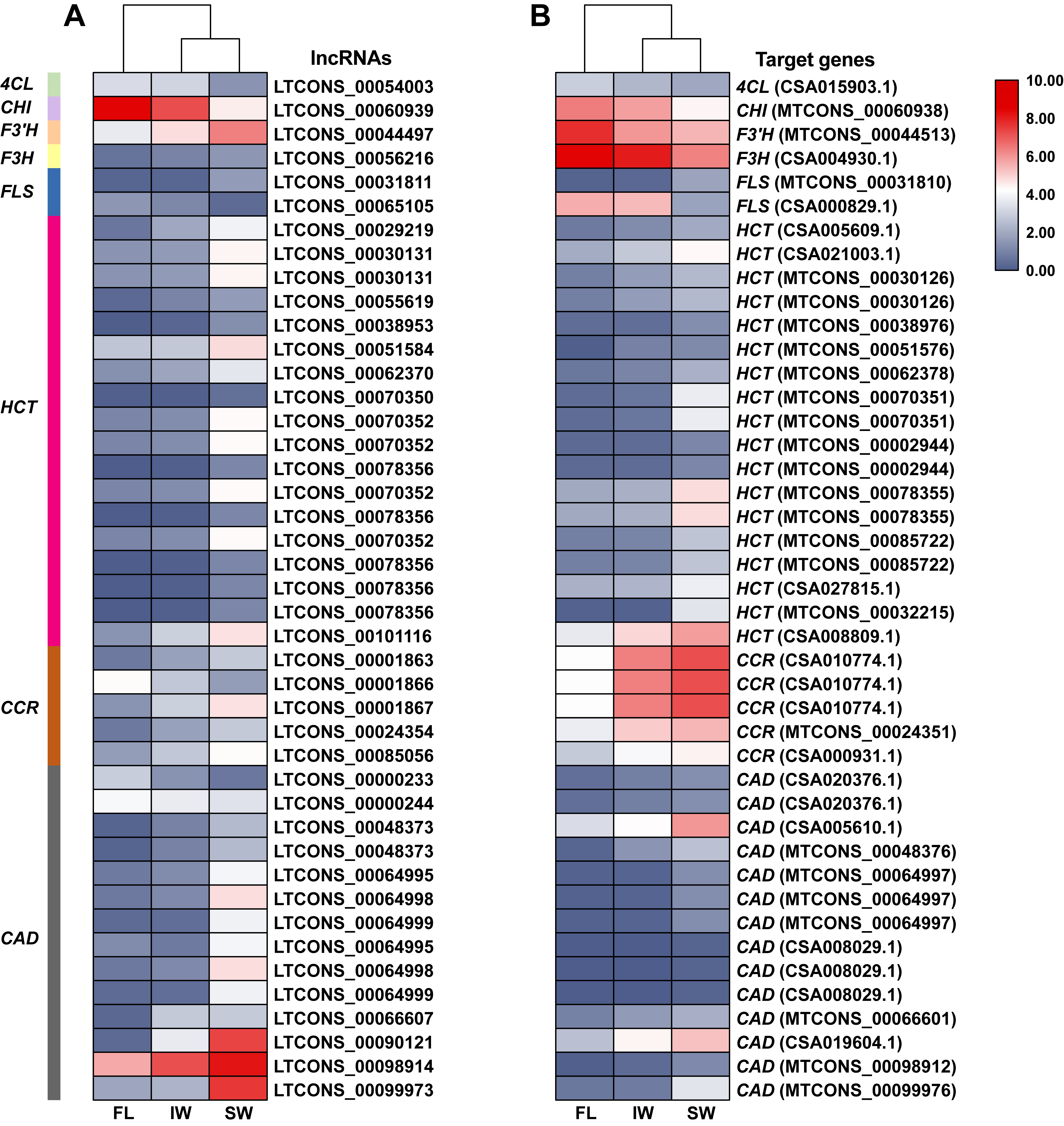

Supplement: Supplementary file 4 [file Image_4.tif]

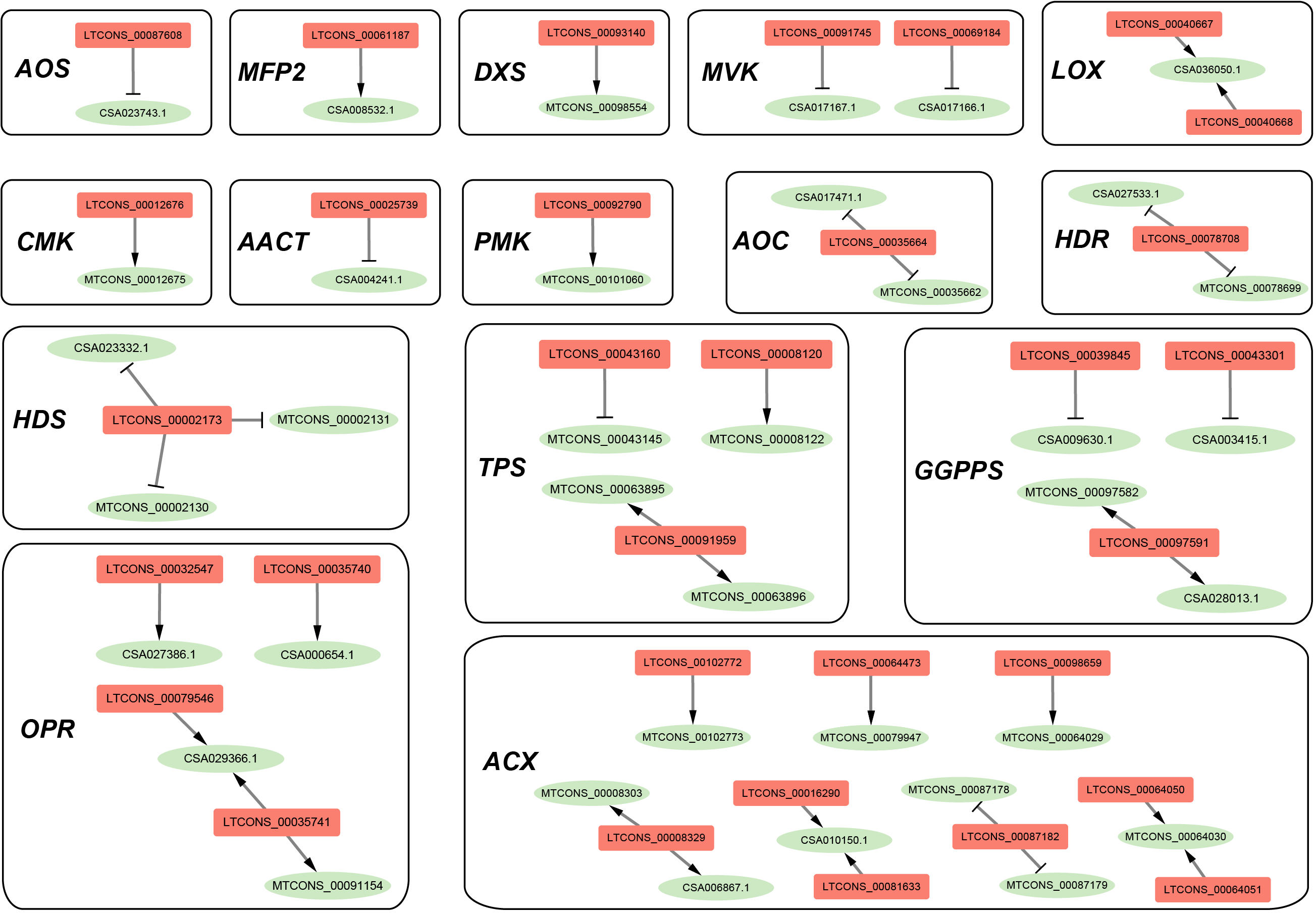

Supplement: Supplementary file 6 [file Image_6.tif]

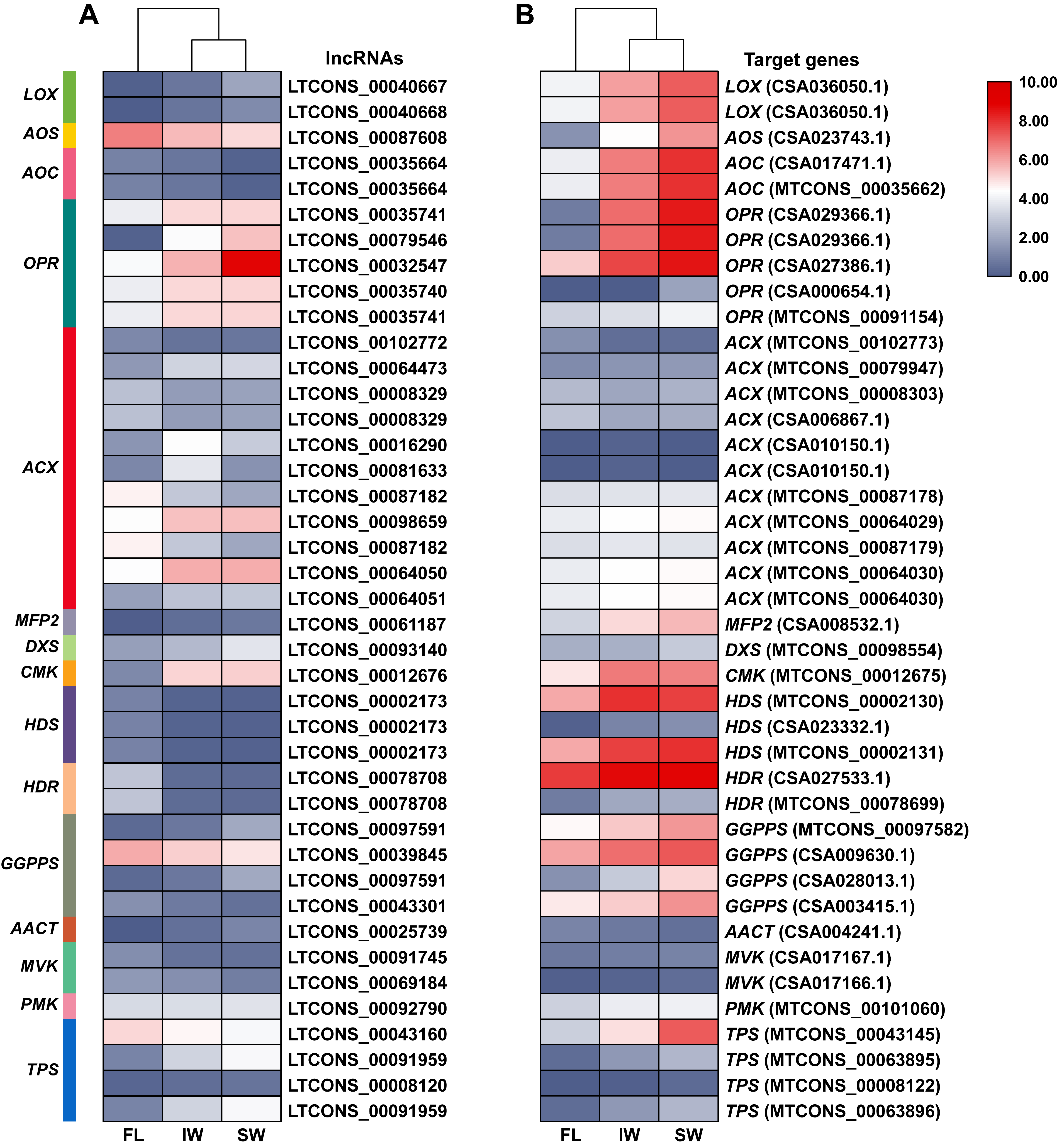

Supplement: Supplementary file 7 [file Image_7.tif]

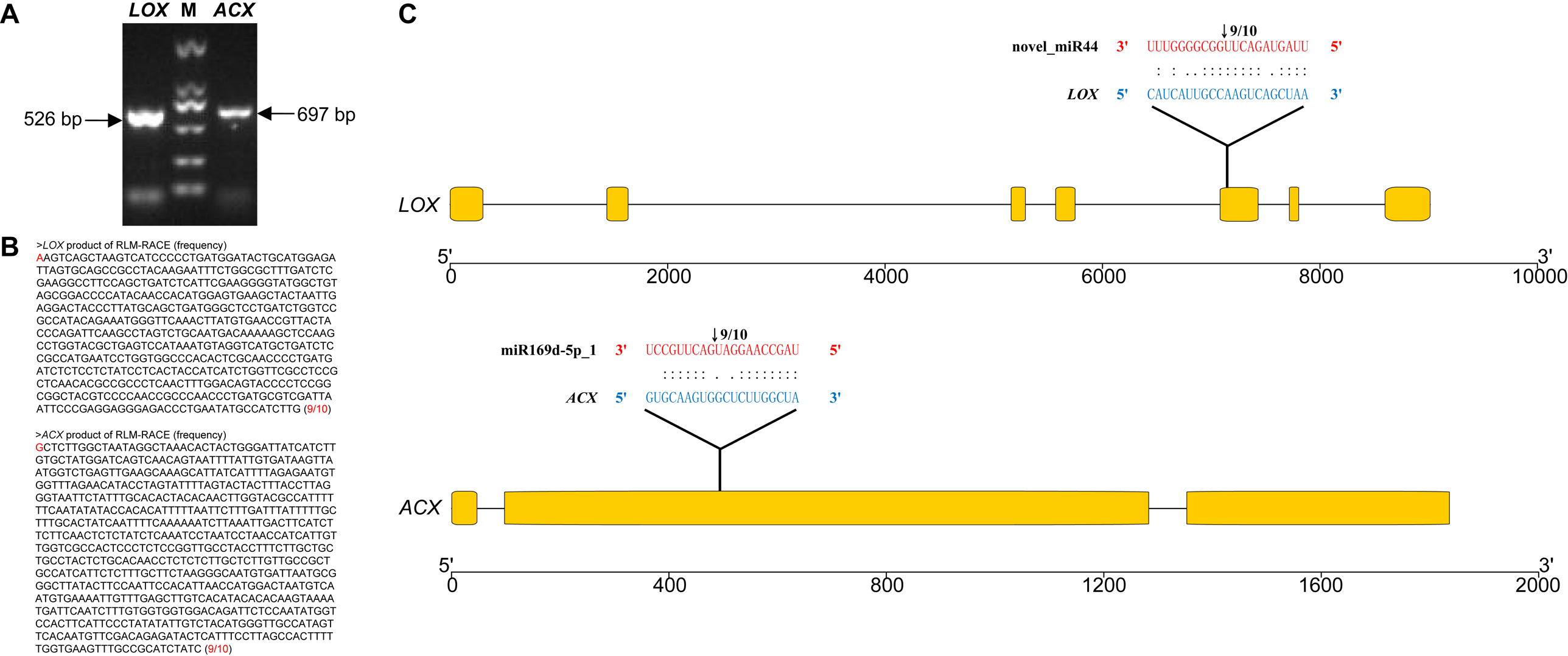

Supplement: Supplementary file 8 [file Image_8.tif]

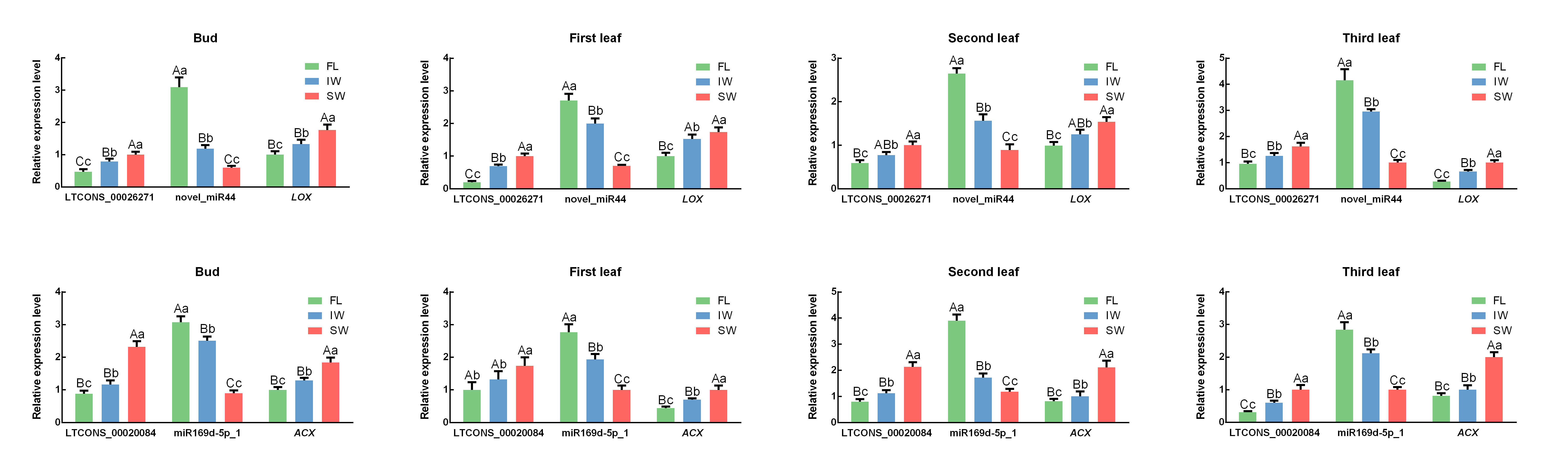

Supplement: Supplementary file 9 [file Image_9.tif]
